# The armed oncolytic adenovirus ZD55-IL-24 eradicates melanoma by turning the tumor cells from the self-state into the nonself-state besides direct killing

**DOI:** 10.1038/s41419-020-03223-0

**Published:** 2020-11-30

**Authors:** Hai-Jun Hu, Xiu Liang, Hai-Lang Li, Chun-Ming Du, Jia-Li Hao, Huai-Yuan Wang, Jin-Fa Gu, Ai-Min Ni, Lan-Ying Sun, Jing Xiao, Jin-Qing Hu, Hao Yuan, Yan-Song Dai, Xiao-Ting Jin, Kang-Jian Zhang, Xin-Yuan Liu

**Affiliations:** 1 State Key Laboratory of Cell Biology, Shanghai Institute of Biochemistry and Cell Biology, Center for Excellence in Molecular Cell Science, Chinese Academy of Sciences, 200031 Shanghai, China; 2 University of Chinese Academy of Sciences, 100049 Beijing, China; 3 School of Life Sciences and Technology, Tongji University, 200092 Shanghai, China; 4 Department of Pharmacy, Xiamen Medical College, 361023 Xiamen, China; 5 Xinyuan Institute of Medicine and Biotechnology, Zhejiang Sci-Tech University, 310018 Hangzhou, China

**Keywords:** Cancer immunotherapy, Drug development

## Abstract

ZD55-IL-24 is similar but superior to the oncolytic adenovirus ONYX-015, yet the exact mechanism underlying the observed therapeutic effect is still not well understood. Here we sought to elucidate the underlying antitumor mechanism of ZD55-IL-24 in both immunocompetent and immunocompromised mouse model. We find that ZD55-IL-24 eradicates established melanoma in B16-bearing immunocompetent mouse model not through the classic direct killing pathway, but mainly through the indirect pathway of inducing systemic antitumor immunity. Inconsistent with the current prevailing view, our further results suggest that ZD55-IL-24 can induce antitumor immunity in B16-bearing immunocompetent mouse model in fact not due to its ability to lyse tumor cells and release the essential elements, such as tumor-associated antigens (TAAs), but due to its ability to put a “nonself” label in tumor cells and then turn the tumor cells from the “self” state into the “nonself” state without tumor cell death. The observed anti-melanoma efficacy of ZD55-IL-24 in B16-bearing immunocompetent mouse model was practically caused only by the viral vector. In addition, we also notice that ZD55-IL-24 can inhibit tumor growth in B16-bearing immunocompetent mouse model through inhibiting angiogenesis, despite it plays only a minor role. In contrast to B16-bearing immunocompetent mouse model, ZD55-IL-24 eliminates established melanoma in A375-bearing immunocompromised mouse model mainly through the classic direct killing pathway, but not through the antitumor immunity pathway and anti-angiogenesis pathway. These findings let us know ZD55-IL-24 more comprehensive and profound, and provide a sounder theoretical foundation for its future modification and drug development.

## Introduction

Oncolytic viruses are therapeutically useful viruses that selectively infect and damage cancerous tissues without causing harm to normal tissues^1^. ZD55-IL-24, one of the most effective armed oncolytic viruses developed so far, was constructed by cloning the foreign antitumor gene mda-7/interleukin-24 (IL-24) into the tumor-targeting replicative viral vector ZD55^2,3^. ZD55 is an E1B 55-kDa gene-deleted adenovirus that is similar to the ONYX-015, but with the marked difference of a cloning site to insert foreign genes^4^. Another two differences between ZD55 and other oncolytic adenoviruses are that ZD55 is an adenovirus type 5 homozygote and has an intact E3 region, whereas the most of other oncolytic adenoviruses are adenovirus chimaeras (e.g., ONYX-015 is a type 2/5 chimaera and Colo-Ad1 is a type 11/3 chimaera)^1,4,5^ or have major deletions within the E3 region (e.g., ONYX-015, H101, and CG0070)^1,5,6^. Our previous studies have shown that ZD55-IL-24 could selectively replicate in many types of human tumor cells and kill these cells much more effectively than ONYX-015 or the replication-defective adenovirus carrying the IL-24 gene both in vitro and in vivo^2,7–9^. In addition, the greatest limitation of oncolytic virotherapy is that the vast majority of oncolytic viruses cannot be administered systemically (e.g., intravenous injection and intraperitoneal injection), but must be administered intratumorally^1,5,10,11^. Hence, oncolytic viruses are difficult to be used to treat the vast majority of tumors in clinic. To overcome this limitation, we constructed a PEG/Lipids/calcium phosphate (CaP)-OncoAd (PLC-OncoAd) delivery system for ZD55-IL-24 in our recent work^12^. ZD55-IL-24, therefore, has great development prospects. Although our previous work on ZD55-IL-24 has been encouraging, some critical problems still exist. For example, we focused on its application in our past research and hence knew little about its antitumor mechanism^2,7–9,12,13^.

The mechanisms through which oncolytic viruses mediate tumor rejection are incompletely understood^5^. The well-known mechanism is the direct killing pathway. Once attached to and entered tumor cells, oncolytic viruses replicate and assemble to progeny virion in tumor cells, eventually leading to tumor cell lysis and viral progeny release. Not only do oncolytic viruses directly kill tumor cells at the end of their lytic cycle, but also progeny viruses spread throughout a tumor, infecting other cancer cells, further producing more viral progeny and improving their antitumor efficacy^10^. Recently, a study about oncolytic Newcastle disease virus revealed that the localized oncolytic virus infection in tumors could be used to drive systemic antitumor immunity^14^. It suggests that oncolytic viruses mediate antitumor activity also through the antitumor immunity pathway. However, little is known about the mechanism of the antitumor immunity pathway. A prevalent notions is that the oncolytic virus infection can result in immunogenic tumor cell death and release the essential elements such as TAAs including neo-antigens, viral pathogen-associated molecular patterns (PAMPs), cellular danger-associated molecular pattern signals (DAMPs), and cytokines such as type I interferons (IFNs), which result in the generation of antitumor immunity^5,14–16^. Based on the above, it is inadequate to fully describe the antitumor mechanism of ZD55-IL-24 only in immunodeficient mouse model for the lack of a functional immune system, and it is also very significant to study the antitumor mechanism of ZD55-IL-24 in immunocompetent mouse model. In this manuscript, we sought to explore the underlying antitumor mechanism of ZD55-IL-24 both in B16-bearing immunocompetent mouse model and A375-bearing immunocompromised mouse model. These results may help us to better understand the antitumor mechanism of ZD55-IL-24 and have some implications for further oncolytic adenovirus design.

## Materials and methods

### Cells

All the cell lines used in this study were obtained from the Cell Bank of the Type Culture Collection of the Chinese Academy of Sciences (Shanghai, China). HEK293 cell was a human embryonic kidney cell line, transformed with Ad5 E1. B16 and A375 were the murine and human melanoma cell lines. LLC1 and A549 were the murine and human lung carcinoma cell lines. CT26.WT and SW620 were the murine and human colorectal cancer cell lines. Additionally, bEnd.3 cell was a murine vascular endothelial cell line. The B16 and CT26.WT cell lines were cultured in RPMI-1640. The SW620 and A549 cell lines were cultured in L15 and F-12K, respectively. Other cell lines were cultured in DMEM. Media were supplemented with 10% FBS, 50 U/mL penicillin, and 50 μg/mL streptomycin. All cell lines were maintained at 37 °C with 5% CO_2_. The frequently used B16 cell line and A375 cell line were not contaminated by mycoplasma, which was confirmed by PCR detection^17^. Other not frequently used cell lines were not performed.

### Adenoviruses

The three recombinant adenoviruses (ZD55, ZD55-EGFP, ZD55-IL-24) used in this study have been previously described^2,4^. Briefly, ZD55 is a conditionally replicating adenovirus type 5 with E1B (55-kDa)-deleted. ZD55-EGFP is a foreign EGFP gene-inserted ZD55 expressing EGFP. ZD55-IL-24 is a foreign human IL-24 gene-inserted ZD55 expressing IL-24. Adenoviruses were propagated in HEK293 cells and purified by CsCl equilibrium centrifugation. Virus titers were determined by TCID50 assay using HEK293 cells and converted to PFU. The titer of each adenovirus was adjusted to 1.5 × 10^10^ PFU/mL, and were administrated intratumorally at 50 μL/dose.

### CCK-8 assay

Cell survivals were determined by the Cell Counting Kit-8 (CCK-8) assay kit (cat#C0038, Beyotime, Shanghai, China) according to the protocol provided by the manufacturer. Briefly, cells were seeded at 1 × 10^4^ cells/well in 96-well plates and the next day infected with ZD55-IL-24 at a serial dilution. At the indicated times (2–4 days after infection according to the growth characteristic of the cell lines), 100 μL medium of each well was removed from the total 200 μL medium, and 10 μL CCK-8 was added to each well. Cells were then incubated at 37 °C 5% CO_2_ for 2–4 h. Absorbance were measured at 450 nm using a Thermo MK3 Microplate Reader.

### ZD55-EGFP infection analysis

B16 cells or A375 cells were seeded at 10^4^ cells/well in 96-well plates and the next day infected with ZD55-EGFP at a serial dilution. The infected cells were determined by direct detection of EGFP fluorescence using an Olympus IX71 inverted fluorescence microscope. Photographs were taken on Day 0, Day 1, Day 2, and Day 4. CCK-8 assay was performed according to the above protocol after the photographs of Day 4 were taken.

### Transmission electron microscopy

All the cell samples collected were fixed with 2.5% glutaraldehyde overnight followed by 1% osmium tetroxide for 1.5 h. The cell samples were then dehydrated using a graded series of ethanol, rinsed with acetone and permeated overnight with embedding buffer. Sections of 70 nm thicknesses were dual-stained with 2% uranyl acetate and lead citrate. Adenovirus particles were examined by transmission electron microscope (FEI, Hillsboro, OR, USA).

### Western blot

The Western blot was performed according to the standard procedures. The primary antibody against IL-24 (cat#K101) was purchased from Genhunter (Nashville, USA). The primary antibody against adenovirus type 2/5 E1A (cat#sc-25) was obtained from Santa Cruz. The primary antibody against adenovirus type 5 Hexon (cat# LF-MA0177) was obtained from Thermo Fisher Scientific. The primary antibody against GAPDH (cat#Ab103) was obtained from Vazyme (Nanjing, China). The secondary antibody (cat#sc-2005) was purchased from Santa Cruz.

### Mouse cytokine antibody arrays

To detect changes of cytokines in tumors after ZD55-IL-24 treatment, the Mouse L308 Array Kit (cat#AAM-BLG-1–4, Ray Biotech) was used following the manufacturer’s instructions. Briefly, tumor tissues were harvested from mice two days after the last injection and lysed using cell lysis buffer. Tumor tissue lysates were biotinylated for 30 min at room temperature. Meanwhile, cytokine array slides were blocked with blocking buffer for 30 min at room temperature. Slides were then incubated with the biotin-labeled tumor tissue lysates containing equal amounts of protein overnight at 4 °C, washed and incubated with Cy3-conjugated streptavidin for 2 h at room temperature. Unbound Cy3-conjugated streptavidin was washed out with wash buffer. The signals were scanned using a GenePix 4000B microarray scanner (Axon) and analyzed using the software GenePix Pro 6.0. Details of the microarray design and data analysis are available in manufacturer’s instructions.

### Immunohistochemistry

Tumor tissues were harvested from mice 2 days after the last injection, embedded in OCT (Leica) and cut into 8 µm cryosections on slides. Subsequently, samples were fixed with ice cold acetone for 15 min at −20 °C, and permeabilized for 5 min with 0.5% Triton X-100 in phosphate-buffered saline (PBS) (Ki-67 assay) or not. Blocking was achieved using 10% horse serum and 1% BSA in PBS for 1 h at room temperature. Tissues were stained at 4 °C overnight using the appropriate antibodies diluted in blocking solution. Antibodies used for immunohistochemistry were purchased from Biolegend (CD8a PE, cat#100708; NK 1.1 Alexa Fluor^®^ 647, cat#108720; Ly-6G/Ly-6C (Gr-1) Alexa Fluor^®^ 488, cat# 108417, and their isotype control) and eBioscience (Ki-67 FITC, cat#652410; CD31 FITC, cat#11-0311-81; CD11b eFluor 660, cat#50-0112-80, and their isotype control). Apoptotic cells in the tumor tissue sections were detected using a TUNEL assay kit (cat#C1089, Beyotime), and the staining was performed according to the manufacturer’s recommendations. Nuclear counterstaining was performed using DAPI. Slides were mounted with ProLong gold antifade mountant (cat#P36930, Thermo Fisher Scientific). Images were captured using Olympus BX51 upright microscope.

### Flow cytometry

Cells cultured in vitro or isolated from tumors or spleens were processed for surface labeling with appropriate antibodies. Propidium iodide was used to distinguish the live cells. Cells were further permeabilized using 70% ethanol or True-Nuclear™ transcription factor buffer set (cat# 424401, BioLegend) and stained for Ki-67 or FOXP3. Data were acquired using the Beckman CytoFLEX flow cytometer and analyzed using FlowJo software. Antibodies used for flow cytometry were purchased from Biolegend (anti-mouse CD16/32, cat#101320; CD8a Alexa Fluor^®^ 647, cat#100724; NK 1.1 Alexa Fluor^®^ 647, cat#108720; Ly-6G/Ly-6C (Gr-1) Alexa Fluor^®^ 488, cat#108417; CD19 FITC, cat#152404; CD45R/B220 Alexa Fluor^®^ 647, cat#103226; F4/80 Alexa Fluor^®^ 488, cat#123120; CD206 Alexa Fluor^®^ 647, cat#141712; FOXP3 Alexa Fluor^®^ 488, cat#126406; CD4 Alexa Fluor^®^ 647, cat#100530; CD11c Alexa Fluor^®^ 488, cat#117311; H-2K^b^/H-2D^b^ FITC, cat#114606; I-A/I-E Alexa Fluor^®^ 647, cat#107618; CD80 Alexa Fluor^®^ 647, cat#104718; CD86 FITC, cat#105006; CSF-1R Alexa Fluor^®^ 488, cat#135511) and eBioscience (anti-mouse Ki-67 FITC, cat#11-5698-82; CD3ε FITC, cat#11-0031-82; CD11b eFluor 660, cat#50-0112-82).

### Animal experiments

All animal experiments were approved by the Institutional Animal Care and Use Committee of Shanghai Institute of Biochemistry and Cell Biology, Chinese Academy of Sciences. Female C57BL/6 mice 6 weeks of age or BALB/c nude mice 4 weeks of age were obtained from SLAC (Shanghai, China) and quarantined for about 1–2 weeks before tumor implantation. For the single tumor model, an inoculum of 1 × 10^6^ B16 cells or 2 × 10^6^ A375 cells was injected subcutaneously only on the right flank of mice in 100 µL sterile PBS. For the two-tumor-model, an inoculum of 1 × 10^6^ B16 cells or 2 × 10^6^ A375 cells was injected subcutaneously on the right flank of mice in 100 µL sterile PBS on day 0 and an inoculum of 2 × 10^5^ B16 cells or 4 × 10^5^ A375 cells was injected subcutaneously on the left flank of mice in 100 µL sterile PBS on day 4. When the subcutaneous tumor size reached an appropriate volume, the mice were randomized into several groups using R software, and treatments were initiated as indicated in each figure legend. ZD55-IL-24 (7.5 × 10^8^ PFU/dose) or PBS was administrated intratumorally at 50 μL/dose for 5 consecutive days or every other day (for the two-tumor-model, ZD55-IL-24 was administrated only into right flank tumors, but not left flank tumors). Tumor size (volume = length × width^2^ × 0.5) and body weight were measured every 2 days, and mice were euthanized when the average right tumor volume of PBS group exceeded 2000 mm^3^. Animal survival was also recorded every 2 days. Photographs of the tumors resected from the sacrificed mice were taken immediately.

### Depletions

Cellular subsets were depleted by administering 400 µg of depleting antibody i.p. twice weekly beginning 1 day before therapy as indicated: CD8^+^ T cells with anti-CD8α (cat#BE0061, BioXCell), CD4^+^ T cells with anti-CD4 (cat#BE0003-1, BioXCell), NK cells with anti-NK1.1 (cat#BE0036, BioXCell), neutrophils with anti-Ly-6G (cat#BE0075-1, BioXCell). Macrophages were depleted using 300 µg anti-CSF-1R (cat#BE0213, BioXCell) every other day. Cellular depletions of CD8^+^ T cells, CD4^+^ T cells, NK cells, neutrophils, and macrophages were confirmed by flow cytometry of peripheral blood mononuclear cells (PBMCs) (Supplementary Fig. 5A).

### Cytotoxicity assay

B16 cells were seeded at 1 × 10^4^ cells/well in 96-well plates and the next day infected with 100 MOI (PFU/cell) ZD55-IL-24 or not. PBMCs were obtained from PBS or ZD55-IL-24-treated mice (C57BL/6 mice without receiving tumor implantation) and cocultured with the above B16 cells at the ratios of 100:1 for 4 h. The killing effect of PBMCs was assayed using the LDH Cytotoxicity Assay Kit (cat#C0017, Beyotime) according to the manufacturer’s instructions.

### Statistical analyses

Statistical analyses were performed using GraphPad Prism 6.0. Comparisons between two groups were performed using Student’s *t*-test. Comparison of multiple groups was performed by analysis of variance (ANOVA). Survival curves were analyzed using log-rank (Mantel–Cox) test. Differences were considered significant at *P* < 0.05 (*P < 0.05, **P < 0.01, ***P < 0.001, ****P < 0.0001, NS, not significant).

## Results

### ZD55-IL-24 inhibits melanoma growth in B16-bearing immunocompetent mouse model not through the classic direct killing pathway, but through unknown indirect pathway

**Fig. 1:**
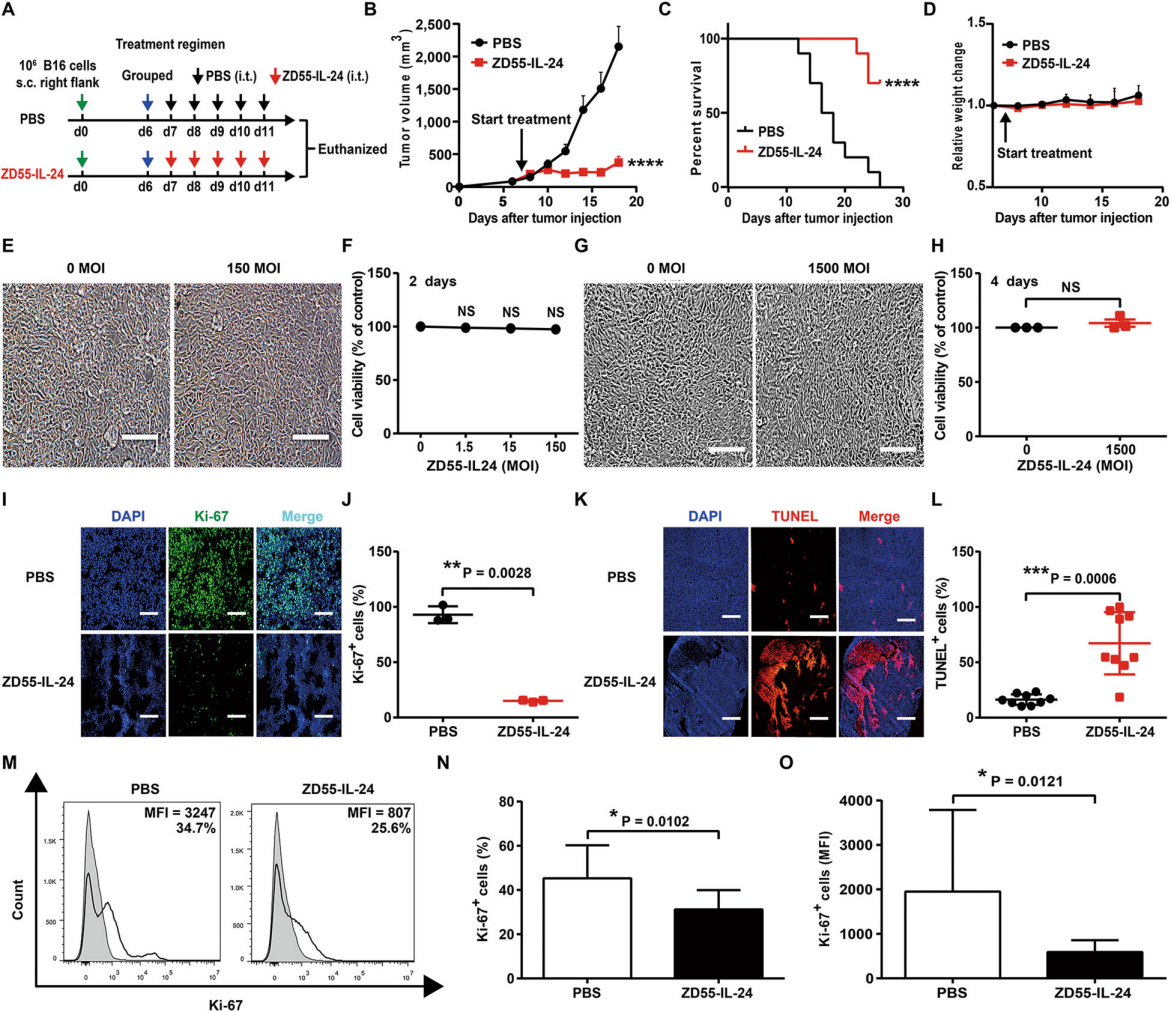
ZD55-IL-24 inhibits tumor growth in B16-bearing immunocompetent mouse model not through the classic direct killing pathway, but through unknown indirect pathway. **A**–**D** The antitumor efficacy of ZD55-IL-24 in B16-bearing immune-competent mouse model. **A** Timeline of tumor engraftment and treatments. **B** Tumor growth curves and **C** survival over time for mice inoculated with 10^6^ B16 melanoma cells s.c. in the right flank and treated 7 days later (the average tumor volume was about 80 mm^3^) with the indicated PBS or ZD55-IL-24. **D** Body weight changes of the B16 melanoma-bearing mice monitored during the therapy period. s.c. subcutaneous injection, i.t. intratumoral injection. Data are presented as the mean ± SEM. *n* = 10 mice per group per experiment. **E**–**H** The B16 cell killing effect of ZD55-IL-24 in vitro. **E** B16 cells were infected with ZD55-IL-24 at a series of MOI (PFU/cell) from 0 to 150, the appearance of cytopathic effect was monitored under microscope, and representative photographs were taken at day 2 post-infection **F** and cell viability was measured by CCK-8 assay. **G** The B16 cells at a density of 10^4^ cells/well cultured in 96-well plates were infected with 1500 MOI (PFU/cell) ZD55-IL-24, the appearance of cytopathic effect was monitored under microscope, and representative photographs were taken at 4 days later, and **H** cell viability was examined by CCK-8 assay. Results represent mean ± SEM of triplicate experiments and are expressed as a percentage of control cells. **I**–**O** The B16 cell killing effect of ZD55-IL-24 in vivo. **I** Tumors resected from B16-bearing C57BL/6 mice receiving PBS or ZD55-IL-24 treatment as indicated in **A** were analyzed 2 days after the last injection by immunohistochemical staining. Shown are representative micrographs of tumor sections immunostained for the proliferation marker Ki-67 (green), and **J** quantification of the number of Ki-67^+^ cells in **I** (Ki-67^+^ nuclei as % of total nuclei; *n* = 3). **K** Shown are representative images of tumor sections immunostained for the apoptosis marker TUNEL (red), and **L** quantification of the TUNEL^+^ cells in **K** (TUNEL^+^ cells as % of total cells; *n* = 9). Nuclei is counterstained with DAPI (blue). Data are mean ± SD. Scale bars, 300 µm. Shown is one of three independent experiments. **M** C57BL/6 mice with B16 tumors were treated as indicated in Fig. 4**A**, and then the tumor cells were isolated for flow cytometry analysis. Shown are representative flow cytometry plots for Ki-67, and **N** percentages or **O** average median fluorescent intensities (MFI) of Ki-67^+^ cells, mean ± SEM is shown. Data represent cumulative results from eight independent experiments.

In an attempt to evaluate the impact of ZD55-IL-24 on the immune system, we here used it to treat C57BL/6 mice bearing B16 melanoma (Fig. 1A). Originally it was thought that ZD55-IL-24 would have greatly reduced or even no efficacy in immunocompetent mouse-murine tumor model for the known no replication of human adenovirus serotype 5 in murine cells. To our surprise, ZD55-IL-24 also induced robust tumor inhibition (Fig. 1B) and greatly prolonged the shortened lifespan of mice (Fig. 1C) in B16-bearing immunocompetent mouse model. Of note, despite high anti-melanoma efficacy, ZD55-IL-24 therapy was associated with no significant systemic toxicity, as mice did not show shortened lifespan and weight or hair loss (Fig. 1D).

To further understand the cellular and molecular mechanisms underlying the observed therapeutic effect of ZD55-IL-24 in B16-bearing immunocompetent mouse model, we first analyzed the direct killing effect of ZD55-IL-24 in B16 cells in vitro. ZD55-IL-24 was unable to induce obvious tumor-selective cytotoxicity in B16 cells in vitro (Fig. 1E, F). Even though the multiplicity of infection (MOI) was increased to a very high level and the treatment time was extended to the maximum, no significant cytopathic effects were observed in B16 cells too (Fig. 1G, H). Our further results showed that ZD55-IL-24 could effectively kill human tumor cells in vitro, though it was not observed in matched murine tumor cells (Supplementary Fig. 1A–H), excluding the possibility of the ZD55-IL-24 we used were inactive. We proceeded to evaluate whether ZD55-IL-24 therapy induced B16 cell death in vivo. Intriguingly, although ZD55-IL-24 could not kill B16 cells in vitro, it could robustly inhibit proliferation (Fig. 1I, J, M–O) and greatly promote apoptosis (Fig. 1K, L) in B16 cells within tumor tissues, suggesting that ZD55-IL-24 inhibited melanoma growth in B16-bearing immunocompetent mouse model not through the well-known direct pathway of inducing tumor-selective cytotoxicity, but through an unknown indirect pathway.

### ZD55-IL-24 therapy remodels the cytokine microenvironment of the established tumors in B16-bearing immunocompetent mouse model

**Fig. 2:**
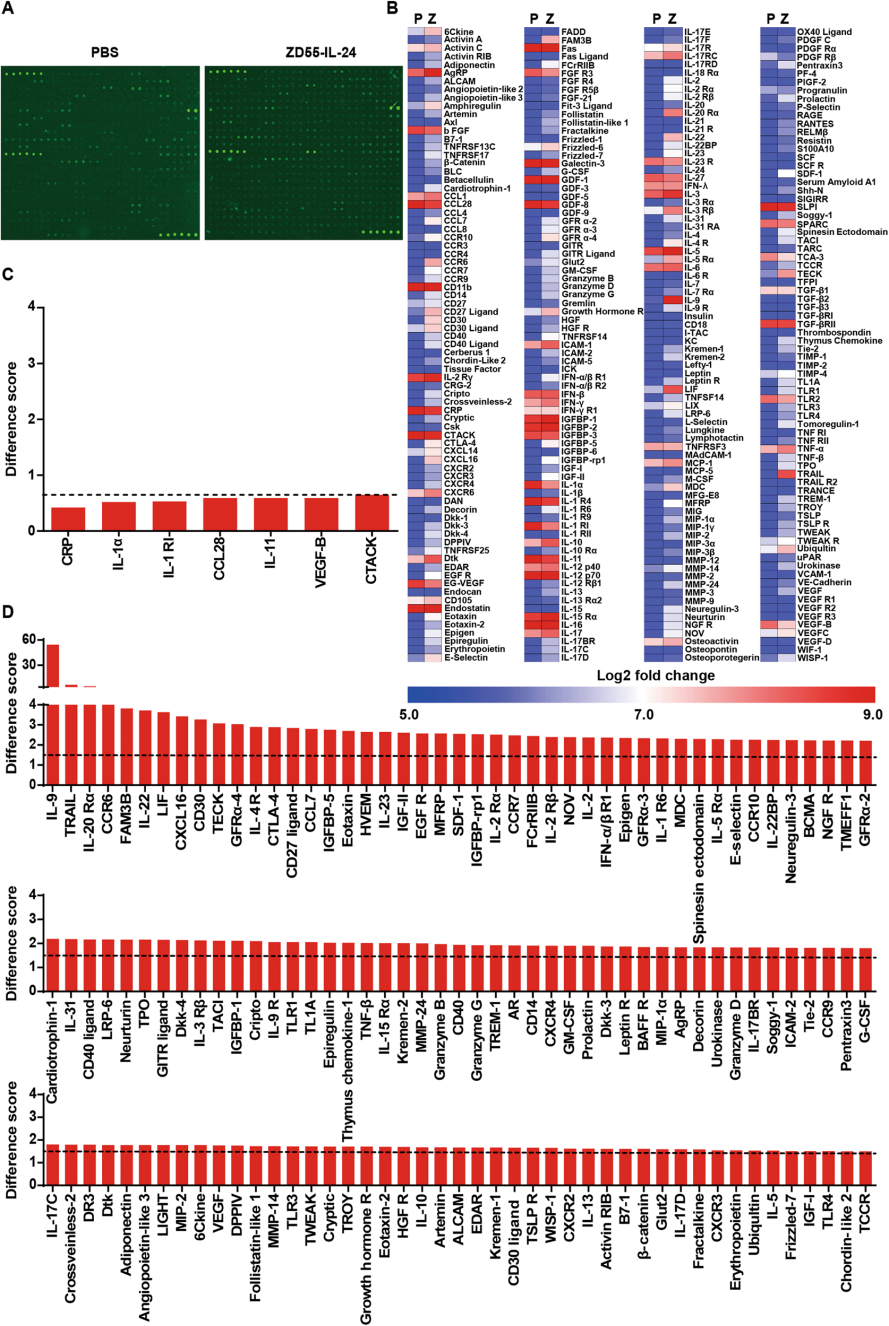
ZD55-IL-24 therapy remodels the cytokine microenvironment of the established tumors in B16-bearing immunocompetent mouse model. C57BL/6 mice bearing B16 tumors were treated with the regimens indicated in Fig. **A**. Two days after the last injection, tumors were isolated and cytokine levels were measured by cytokine antibody arrays. **A** Original images of cytokine antibody arrays. **B** Heat-map of cytokine changes in **A** (average signal intensity of two repeat spot) (see also Supplementary Table 1). P PBS, Z ZD55-IL-24. **C** The down-regulated cytokines in **B**. Difference scores ≤0.65 (dashed line) are considered as significant down-regulation. **D** The up-regulated cytokines in **B**. Difference scores ≥1.5 (dashed line) are considered as significant up-regulation.

To further determine the indirect pathway through which ZD55-IL-24 inhibited melanoma growth in B16-bearing immunocompetent mouse model, we looked for the changes of 308 mouse cytokines that correlated closely with tumor rejection within tumors using cytokine antibody array analysis. Seven cytokines were significantly downregulated (Fig. 2A–C and Supplementary Table 1) and up to 135 cytokines were significantly upregulated (Fig. 2A, B, D and Supplementary Table 1). The most upregulated cytokine was IL-9 which increased up to 54 times, and the most downregulated cytokine was C-reactive protein (CRP) which decreased about two times. Of note, it has been reported that the inflammation marker CRP was an independent prognostic marker in patients with melanoma^18^. Elevated CRP has been correlated with poor prognosis in melanomas. Hence, the decrease of CRP level in ZD55-IL-24-treated mice suggested the good prognosis of ZD55-IL-24 therapy at the molecular level.

### ZD55-IL-24 eliminates established melanoma in B16-bearing immunocompetent mouse model mainly through the indirect pathway of inducing systemic antitumor immunity

**Fig. 3:**
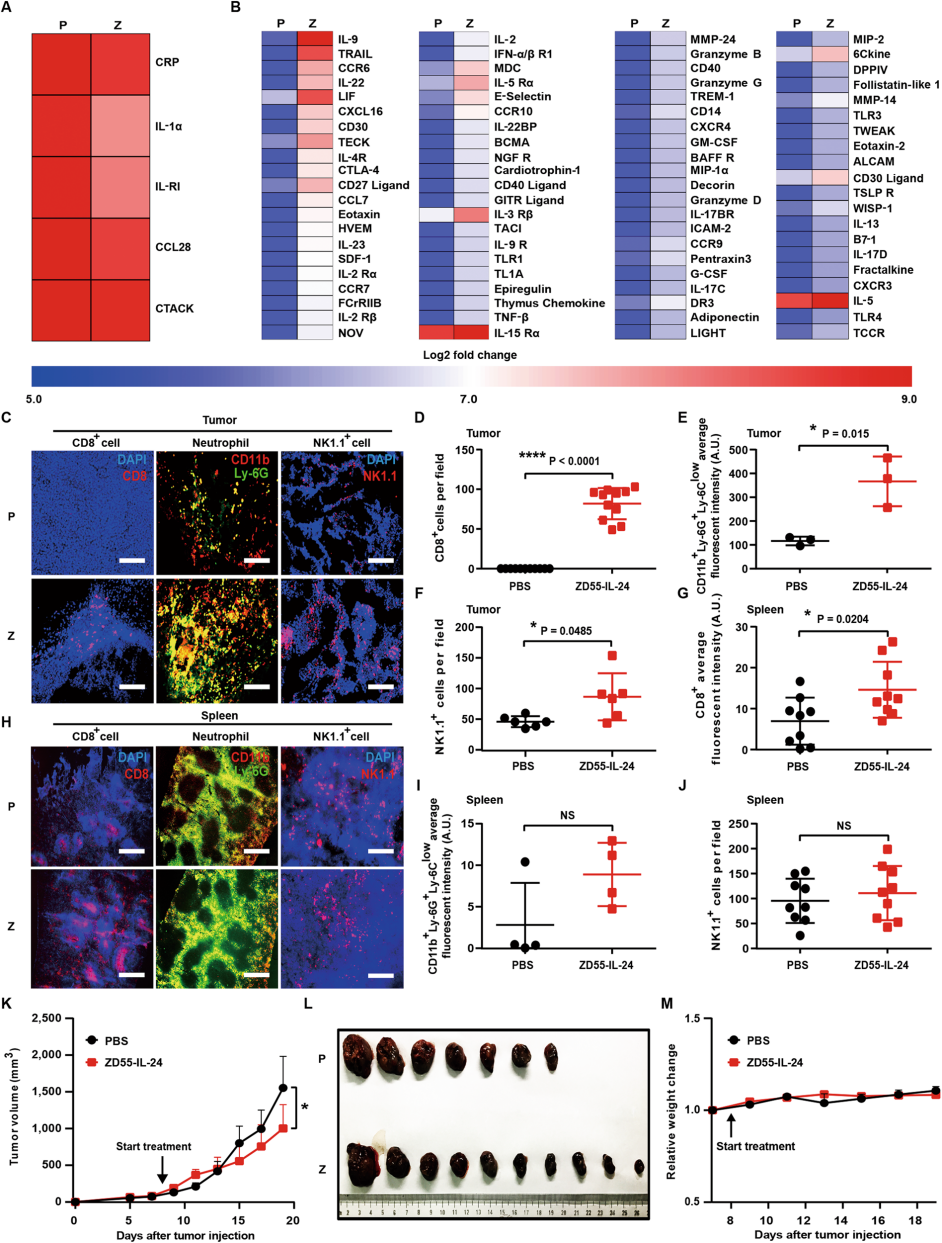
ZD55-IL-24 inhibits melanoma growth in B16-bearing immunocompetent mouse model mainly through the indirect pathway of inducing systemic antitumor immunity. **A**, **B** Changes of the immune-related cytokines in tumors treated with ZD55-IL-24. **A** Heat-map of the down-regulated immunosuppressive cytokines in Fig. 2**C**. **B** Heat-map of the up-regulated immunostimulatory cytokines in Fig. 2**D**. **C**–**J** Immunohistochemical analysis of immune cell infiltration in tumors, as well as recruitment and activation in spleens. Tumors and spleens resected from B16-bearing C57BL/6 mice receiving PBS or ZD55-IL-24 treatment indicated in Fig. 1**A** were analyzed 2 days after the last injection by immunohistochemical staining. **C** Shown are representative images of tumor sections immunostained for CD8 (red, left panel), CD11b and Ly-6G (yellow, middle panel), and NK1.1 (red, right panel). **D** Quantification of the CD8^+^ cells in **C** (*n* = 11). **E** Quantification of the CD11b^+^Ly-6G^+^Ly-6C^low^ neutrophils in **C** (*n* = 3). **f** Quantification of the NK1.1^+^ cells in **c** (*n* = 6). **G** Quantification of the CD8^+^ cells in **H** (*n* = 9). **H** Shown are representative images of spleen sections immunostained for CD8 (red, left panel), CD11b and Ly-6G (yellow, middle panel), and NK1.1 (red, right panel). **I** Quantification of the CD11b^+^Ly-6G^+^Ly-6C^low^ neutrophils in **H** (*n* = 3). **j** Quantification of NK1.1^+^ cells in **h** (*n* = 9). Nuclei is counterstained with DAPI (blue). Data are mean ± SD. Scale bars, 300 µm. Shown is one of three independent experiments. **K**–**M** The anti-melanoma efficacy of ZD55-IL-24 in B16-bearing immunocompromised mouse model. BALB/c nude mice were inoculated with B16 tumors and treated with PBS or ZD55-IL-24 as indicated in Fig. 1**A**. **K** Shown are tumor growth curves and **L** photograph of tumors resected from the sacrificed mice at the end of the experiment. **M** Body weight changes of the mice monitored during the therapy period. P PBS, Z ZD55-IL-24, mean ± SEM is shown. *n* = 7 mice for PBS-treated group, and *n* = 10 mice for ZD55-IL-24-treated group. Data represent results from one of two independent experiments.

Through analysis of the above down-regulated and up-regulated cytokines, we noticed that five down-regulated cytokines had a function of suppressing antitumor immunity (Fig. 3A), and 83 up-regulated cytokines had a function of promoting antitumor immunity (Fig. 3B), hinting that ZD55-IL-24 probably inhibited tumor growth in B16-bearing immunocompetent mouse model through inducing antitumor immunity. Given the critical role of CD8^+^ T cells, neutrophils, and NK cells in antitumor immunity^19^, we thus analyzed the infiltration of these immune cells in tumors, as well as recruitment and activation of these cells in spleens. As expected, immunohistochemical analysis results revealed that CD8^+^ T cells, CD11b^+^Ly-6G^+^Ly-6C^low^ neutrophils, and NK cells were present at high density throughout the ZD55-IL-24-treated tumors, with less effective PBS treatment eliciting fewer CD8^+^ T cells, CD11b^+^Ly-6G^+^Ly-6C^low^ neutrophils, and NK cells in the bulk tumor mass (Fig. 3C–F), demonstrating that ZD55-IL-24 inhibited melanoma growth in B16-bearing immunocompetent mouse model through the indirect pathway of inducing antitumor immunity.

The spleen, where mature naive lymphocytes are maintained, collects antigen from the blood and initiates the systemic immune responses^20^. Notably, although the number of CD11b^+^Ly-6G^+^Ly-6C^low^ neutrophils and NK cells almost unchanged (Fig. 3H–J), a significant increased number of CD8^+^ T cells were observed in the spleens of ZD55-IL-24-treated mice (Fig. 3G, H), indicating that ZD55-IL-24 treatment induced systemic antitumor immunity more than local antitumor immunity in tumors. Indeed, our further results demonstrated that ZD55-IL-24 kill not only local tumors (right ZD55-IL-24-injected tumors) but also distant tumors (left ZD55-IL-24-uninjected tumors) in B16-bearing immunocompetent mouse model (Supplementary Fig. 2A–F). In addition, we found that administration of ZD55-IL-24 to B16-bearing nude mice, which lack adaptive immune responses, led to greatly decrease of its anti-melanoma efficacy (Figs. 1A–D, 3K–M), further suggesting that the adaptive antitumor immunity was critical for the therapeutic effect of ZD55-IL-24 in B16-bearing immunocompetent mouse model. Moreover, our further results also showed that ZD55-IL-24 was able to cure small established melanomas (the tumor lesions were just visible) and establish protective immunological memory in cured mice (Supplementary Fig. 3A–E).

### ZD55-IL-24 induces systemic antitumor immunity in B16-bearing immunocompetent mouse model via an immune recognition-based mechanism

**Fig. 4:**
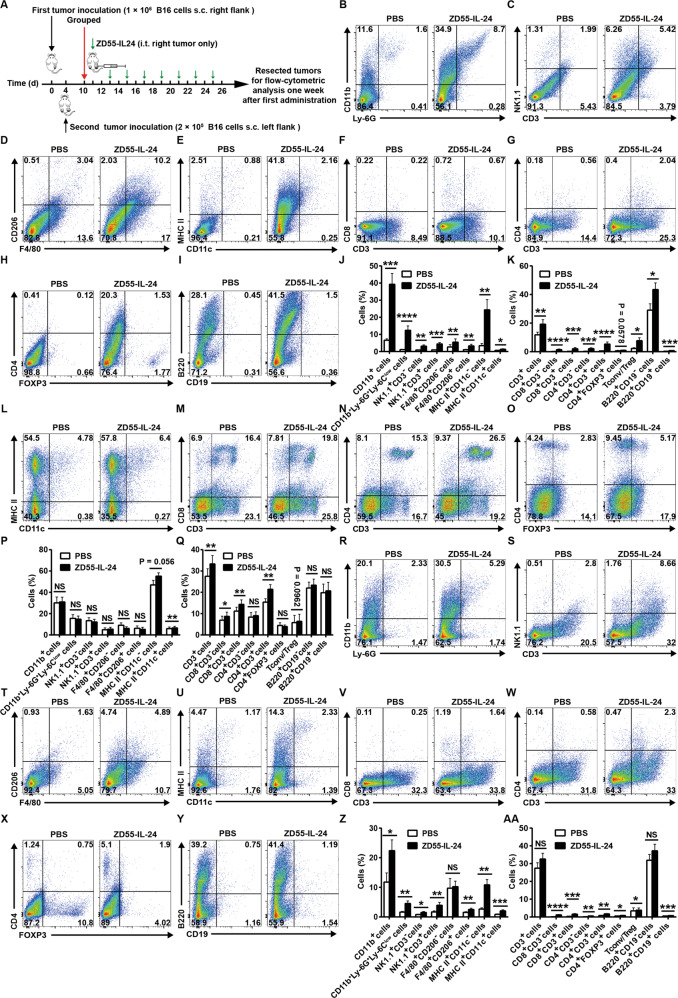
ZD55-IL-24 induces systemic antitumor immunity in B16-bearing immune-competent mouse model by promoting the immune system recognizing tumor cells. **A** Treatment scheme. **B**–**K** Flow-cytometric analysis of immune cell infiltration in right tumors (local ZD55-IL-24-injected tumors). **B** Representative flow cytometry plots of tumor-infiltrating total myeloid cells (CD11b^+^) and neutrophils (CD11b^+^Ly-6G^+^Ly-6C^low^) in right tumors. **C** Representative flow cytometry plots of tumor-infiltrating natural killer cells (NK, NK1.1^+^CD3^−^) and natural killer T cells (NKT, NK1.1^+^CD3^+^) in right tumors. **D** Representative flow cytometry plots of tumor-infiltrating M1 macrophages (F4/80^+^CD206^−^) and M2 macrophages (F4/80^+^CD206^+^) in right tumors. **E** Representative flow cytometry plots of tumor-infiltrating MHC II^+^CD11c^−^ antigen-presenting cells (APCs) and dendritic cells (DCs, MHC II^+^CD11c^+^) in right tumors. **F** Representative flow cytometry plots of tumor-infiltrating total T cells (CD3^+^), CD8^+^CD3^−^ cells, and CD8^+^ T cells (CD8^+^CD3^+^) in right tumors. **G** Representative flow cytometry plots of tumor-infiltrating CD4^+^CD3^−^ cells and CD4^+^ T cells (CD4^+^CD3^+^) in right tumors. **H** Representative flow cytometry plots of tumor-infiltrating conventional T cells (T_conv_, CD4^+^FOXP3^−^) and regulatory T cells (T_reg_, CD4^+^FOXP3^+^) in right tumors. **I** Representative flow cytometry plots of tumor-infiltrating plasma cells (B220^+^CD19^−^) and B cells (B220^+^CD19^+^) in right tumors. **J** Percentages of innate immune cells in right tumors. **K** Percentages of adaptive immune cells in right tumors. **L**–**Q** Flow-cytometric analysis of immune cell recruitment and activation in spleens. **L** Representative flow cytometry plots of MHC II^+^CD11c^−^ APCs and DCs in spleens. **M** Representative flow cytometry plots of total T cells, CD8^+^CD3^−^ cells, and CD8^+^ T cells in spleens. **N** Representative flow cytometry plots of CD4^+^CD3^−^ cells and CD4^+^ T cells in spleens. **O** Representative flow cytometry plots of T_conv_ cells and T_reg_ cells in spleens. **P** Percentages of innate immune cells in spleens. **Q** Percentages of adaptive immune cells in spleens. **R**–**AA** Flow-cytometric analysis of immune cell infiltration in left tumors (distant ZD55-IL-24-uninjected tumors). **R** Representative flow cytometry plots of tumor-infiltrating total myeloid cells and neutrophils in left tumors. **S** Representative flow cytometry plots of tumor-infiltrating NK cells and NKT cells in left tumors. **T** Representative flow cytometry plots of tumor-infiltrating M1 macrophages and M2 macrophages in left tumors. **U** Representative flow cytometry plots of tumor-infiltrating MHC II^+^CD11c^−^ APCs and DCs in left tumors. **V** Representative flow cytometry plots of tumor-infiltrating total T cells, CD8^+^CD3^−^ cells, and CD8^+^ T cells in left tumors. **W** Representative flow cytometry plots of tumor-infiltrating CD4^+^CD3^−^ cells and CD4^+^ T cells in left tumors. **X** Representative flow cytometry plots of tumor-infiltrating T_conv_ cells and T_reg_ cells in left tumors. **Y** Representative flow cytometry plots of tumor-infiltrating plasma cells and B cells in left tumors. **Z** Percentages of innate immune cells in left tumors. **AA** Percentages of adaptive immune cells in left tumors. s.c. subcutaneous injection, i.t. intratumoral injection, mean ± SEM is shown. Data represent cumulative results from nine to twelve (**B**–**K**), seven (**L**–**Q**), or nine (**R**–**AA**) independent experiments.

To understand why ZD55-IL-24 was able to induce systemic antitumor immunity, we further comprehensively analyzed immune cell infiltration in both local and distant tumors, as well as recruitment and activation of these cells in spleens using flow cytometry (Fig. 4A and Supplementary Fig. 4A, B). In agreement with the immunohistochemical results, analysis of the local tumors revealed a robust tumor immune infiltration of both innate and adaptive immune cells. The immune infiltrates were characterized by increase in innate immune compartment, including total myeloid cells, neutrophils, NK cells, NKT cells, M1 macrophages, M2 macrophages, MHC II^+^CD11c^−^ APCs and DCs (Fig. 4B–E, J), and the adaptive compartment, including total T cells, CD8^+^CD3^−^ cells, CD8^+^ T cells, CD4^+^CD3^−^ cells, CD4^+^ T cells, T_reg_ cells, T_conv_/T_reg_, plasma cells, and B cells (Fig. 4F–I, K). In addition, ZD55-IL-24 therapy also led to a significant increase of MHC II^+^CD11c^−^ APCs, DCs, total T cells, CD8^+^CD3^−^ cells, CD8^+^ T cells, CD4^+^ T cells, and T_conv_/T_reg_ in spleens, although it was unable to increase the percentages of other examined immune cells (Fig. 4L–Q), indicating that ZD55-IL-24 treatment induced a systemic antitumor immunity more than local antitumor immunity in tumors, which was also consistent with the above immunohistochemical results. Moreover, analysis of the contralateral tumors revealed a similar increase in the tumor immune infiltration, characterized by increased percentages of all the examined immune cells, with the exception of M1 macrophages, total T cells, and plasma cells (Fig. 4R–AA), further indicating that ZD55-IL-24 treatment induced antitumor immunity not only in local tumors but also in distant tumors. Finally, antibody depletion experiments showed that CD8^+^ T cells were critical to tumor rejection (Supplementary Fig. 5A–D). CD4^+^ T cell, NK cell or neutrophil depletion also led to reductions of efficacy in our two independent experiments, albeit no statistically significant differences. These results indicated that ZD55-IL-24 elicited a substantial immune infiltration in both local and distant tumors with contributions to tumor rejection.

Immune responses are initiated when APCs, particularly DCs, recognize antigens^20^. However, tumor cells tend to be self-origin and are inherently not very immunogenic, hence failure to be recognized by APCs is generally the major obstacle to successfully induce antitumor immunity^20,21^. Of note, the percentages of both MHC II^+^CD11c^−^ APCs and DCs were substantially increased by ZD55-IL-24 therapy in local tumors, spleens, and distal tumors (Fig. 4E, J, L, P, U, Z), suggesting that the primary reason for the capacity of ZD55-IL-24 to induce systemic antitumor immunity in B16-bearing immunocompetent mouse model was attributed to the promotion of the recognition of original host immune system to tumor cells.

### ZD55-IL-24 promotes the immune recognition of tumor cells in B16-bearing immunocompetent mouse model not due to its ability to lyse immunogenic tumor cells and release the essential elements for the induction of antitumor immunity

**Fig. 5:**
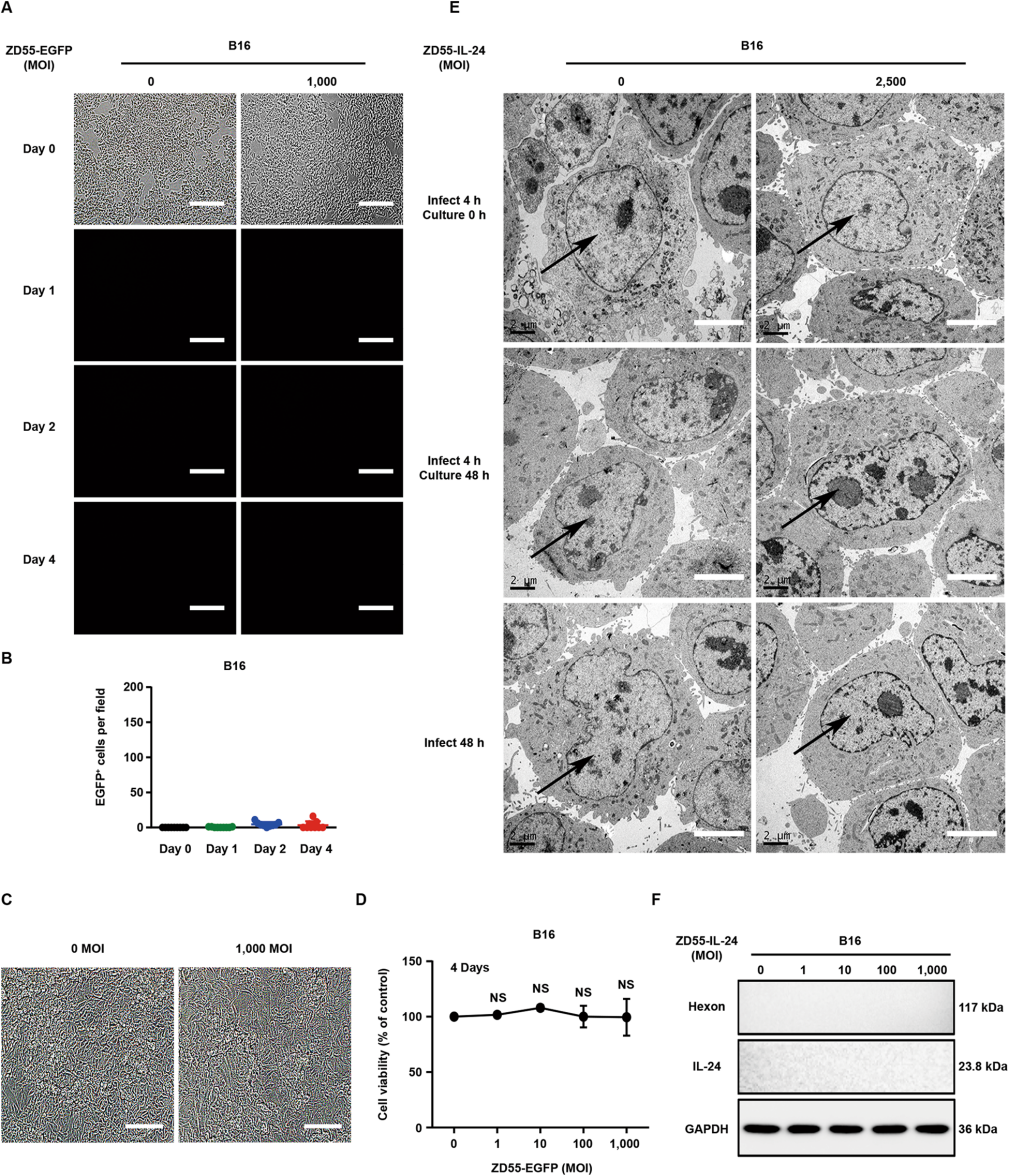
ZD55-IL-24 promotes the immune recognition of tumor cells in B16-bearing immunocompetent mouse model not due to its ability to lyse immunogenic tumor cells and release the essential elements for the induction of antitumor immunity. **A**–**D** Fluorescence microscopic analysis of viral infection and exogenous gene expression in B16 cells. **D** The murine melanoma B16 cells were infected with ZD55-EGFP at a MOI (PFU/cell) of 0 and 1000, and the viral infection and exogenous gene expression were monitored under the fluorescence microscope on Day 0, Day 1, Day 2, and Day 4 after infection. **D** Quantification of the EGFP-positive B16 cells in **A** (*n* = 9). Error bars indicate mean ± SD. Shown is one of three independent experiments. **C** The appearance of cytopathic effect in **A** was monitored under microscope, and representative phase-contrast images were taken at the end of the experiment, and **D** cell viability was measured by CCK-8 assay. Results represent mean ± SEM of triplicate experiments and are expressed as a percentage of control cells. Scale bars, 300 µm. **E** Representative transmission electron microscopy images of B16 cells treated with ZD55-IL-24 at a MOI (PFU/cell) of 0 and 2500. Shown is one of three independent experiments. Nuclei are indicated by the black arrow. Scale bar: 4 μm. **F** Western blot analysis of viral infection and exogenous IL-24 expression in B16 cells infected with ZD55-IL-24 at a series of MOI (PFU/cell) as indicated. Shown is one of three independent experiments.

To further understand why ZD55-IL-24 could promote the original host immune system recognizing tumor cells, we next investigated the changes of B16 cells caused by ZD55-IL-24 treatment. We first examined the viral infection and gene expression of ZD55-IL-24 in B16 cells in vitro. Fluorescence microscopic analysis revealed that ZD55-IL-24 could not successfully infect and express exogenous gene in B16 cells (Fig. 5A–D). In parallel, our transmission electron microscopy and Western blot results also supported this conclusion (Fig. 5E, F), suggesting that ZD55-IL-24 was unable to directly lyse immunogenic tumor cells in B16-bearing immunocompetent mouse model and thus could not release TAAs, PAMPs, DAMPs as well as cytokines, which helped immune system recognizing tumor cells and were thought to be essential for the induction of antitumor immunity by oncolytic viruses^5,14–16^. We thus concluded that ZD55-IL-24 promoted the immune recognition of tumor cells in B16-bearing immunocompetent mouse model not due to its ability to cause immunogenic tumor cell death and release the essential elements for the induction of antitumor immunity. The above results indicated that ZD55-IL-24 induced antitumor immunity in B16-bearing immunocompetent mouse model via a distinct mechanism.

### ZD55-IL-24 promotes the immune recognition of tumor cells in B16-bearing immunocompetent mouse model due to its ability to turn the tumor cells from the “self” state into the “nonself” state

**Fig. 6:**
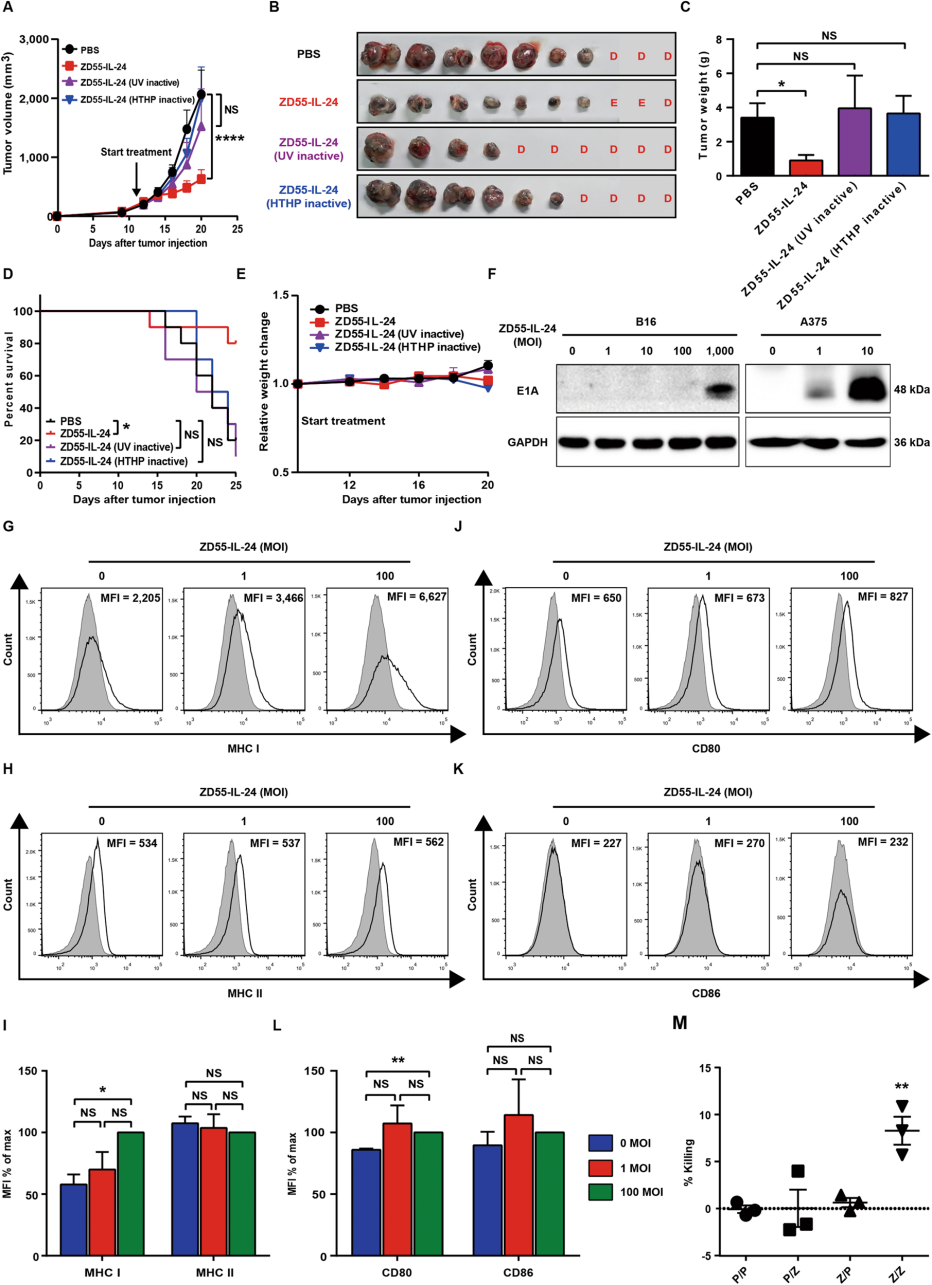
ZD55-IL-24 promotes the immune recognition of tumor cells in B16-bearing immunocompetent mouse model due to its ability to turn the tumor cells from the “self” state into the “nonself” state. **A**–**E** The antitumor efficacy of inactive ZD55-IL-24 in B16-bearing immunocompetent mouse model. C57BL/6 mice were inoculated with B16 tumors and treated with PBS, ZD55-IL-24, or inactive ZD55-IL-24 as indicated in Fig. 4A. **A** In vivo tumor growth curves. **B** Photograph of tumors resected from the sacrificed mice at the end of the experiment. **C** Weight of tumors resected from the sacrificed mice at the end of the experiment. **D** Overall survival. **E** Body weight changes of the mice monitored during the therapy period. UV ultraviolet, HTHP high temperature and high pressure, E eradication, D death. Data represent results from one of two independent experiments with *n* = 10 per group, mean ± SEM is shown. **F** Western blot analysis of E1A expression in B16 and A375 cells infected with ZD55-IL-24 at a series of MOI (PFU/cell) as indicated. Shown is one of three independent experiments. **G**–**I** Flow-cytometric analysis of MHC molecules on the surface of B16 cells infected with ZD55-IL-24 at a MOI (PFU/cell) of 0, 1, and 100. **G** Representative flow cytometry plots of surface MHC I. **H** Representative flow cytometry plots of surface MHC II. **I** MFI in **G** and **H**. **J**–**L** Flow-cytometric analysis of costimulatory molecules on the surface of B16 cells infected with ZD55-IL-24 at a MOI (PFU/cell) of 0, 1, and 100. **J** Representative flow cytometry plots of surface CD80. **K** Representative flow cytometry plots of surface CD86. **L** MFI in **J** and **K**, mean ± SEM is shown. Data represent cumulative results from three independent experiments. **M** Cytotoxicity analysis of PBMCs obtained from ZD55-IL-24-treated mice bearing no tumors to ZD55-IL-24-treated B16 cells in vitro. B16 cells were treated with PBS or 100 MOI (PFU/cell) ZD55-IL-24, and then cocultured with the PBMCs obtained from PBS or ZD55-IL-24-treated mice (C57BL/6 mice without receiving tumor inoculation) at the effector:target ratio of 100:1 to assess the in vitro cytotoxicity. P/P PBS-treated B16 cells cocultured with PBMCs from PBS-treated mice, P/Z PBS-treated B16 cells cocultured with PBMCs from ZD55-IL-24-treated mice, Z/P ZD55-IL-24-treated B16 cells cocultured with PBMCs from PBS-treated mice, Z/Z ZD55-IL-24-treated B16 cells cocultured with PBMCs from ZD55-IL-24-treated mice, mean ± SEM is shown. Data represent cumulative results from three independent experiments.

As the above data showed, it seems that the antitumor immunity inducing process of ZD55-IL-24 has nothing to do with the viral infection of tumor cells. We thus assessed whether the HTHP-inactivated ZD55-IL-24 that completely lost the ability of viral attachment and entry was still able to induce antitumor immunity (Supplementary Fig. 6A–F). Of interest, once lost the capability of viral attachment and entry, ZD55-IL-24 was no longer able to induce antitumor immunity (Fig. 6A–E), indicating that the antitumor immunity inducing process of ZD55-IL-24 in B16-bearing immunocompetent mouse model was dependent on viral attachment and entry. As such, we next examined if ZD55-IL-24 was able to enter B16 cells. We noticed that the anti-melanoma efficacy of wild-type adenovirus type 5 (Ad5.WT) was much higher than ZD55 which was the viral vector of ZD55-IL-24 and was identical to Ad5.WT with the exception of E1B 55-kDa gene deletion (Supplementary Figs. 7B and 8A–E), indicating that the E1B 55-kDa gene played an important role in B16-bearing immunocompetent mouse model. The E1B 55-kDa gene, however, must be expressed in cells to play a role, suggesting that ZD55-IL-24 viral particles were probably able to enter B16 cells. Indeed, although the expression of Hexon and IL-24 was undetectable (Fig. 5F), the expression of E1A was detected in ZD55-IL-24-treated B16 cells in vitro using Western blot **(**Fig. 6F), directly demonstrating that ZD55-IL-24 viral particles could in fact enter B16 cells. These data indicated that ZD55-IL-24 was able to put a “nonself” label into tumor cells which were considered as “self” by original host immune system^20,21^.

Motivated by the above findings, we thus speculated that the capability of ZD55-IL-24 to promote immune system recognizing tumor cells in B16-bearing immunocompetent mouse model was attributed to its ability to turn the tumor cells from the “self” state into the “nonself” state. Indeed, our flow-cytometric results confirmed that ZD55-IL-24 viral infection was able to result in surface up-regulation of major histocompatibility complex (MHC) I, which collected viral antigen in the cytosol and displayed viral antigen on the surface of infected cells^20^ (Fig. 6G–I), indicating that ZD55-IL-24 viral infection was most likely to result in “nonself” viral antigen epitopes presenting on MHC I molecules located on the surface of tumor cells. Furthermore, we also found that ZD55-IL-24 viral infection was able to result in surface up-regulation of CD80 which was one of the major co-stimulatory molecules for T cells^20^ (Fig. 6J–L), further facilitating the induction of antitumor immunity. Finally, we found that the PBMCs obtained from ZD55-IL-24-treated mice (mice without receiving tumor inoculation) could specifically kill ZD55-IL-24-treated B16 cells in vitro (Fig. 6M), indicating that the anti-viral specific immune response induced by ZD55-IL-24 could act specifically against the tumor cells infected with ZD55-IL-24, indirectly demonstrating that ZD55-IL-24 viral infection could result in the presentation of “nonself” viral epitopes on the surface of tumor cells. Taken together, these data indicated that ZD55-IL-24 could put a “nonself” label into tumor cells, and then turn the tumor cells from the “self” state into the “nonself” state, eventually promote the immune system recognizing tumor cells. Importantly, we simultaneously noticed that the UV-inactivated ZD55-IL-24 that has the ability of viral attachment and entry, but has a damaged viral DNA, also failed to induce antitumor immunity (Fig. 6A–E), further suggesting that the “nonself” state turning process of tumor cells induced by ZD55-IL-24 was dependent on the action of viral DNA in tumor cells.

### The exogenous IL-24 gene harbored in ZD55-IL-24 viral genome has no significant contribution to the anti-melanoma efficacy of ZD55-IL-24 in B16-bearing immunocompetent mouse model

In our previous investigation, we have confirmed that the exogenous IL-24 gene harbored in ZD55-IL-24 viral genome played an important role in immunocompromised mouse–human tumor xenograft models^7,8^. Thus, we next evaluated the contribution of exogenous IL-24 gene in B16-bearing immunocompetent mouse model. As mentioned above, our fluorescence microscopy, transmission electron microscopy and Western blot results showed that ZD55-IL-24 could not successfully infect and express exogenous IL-24 gene in B16 cells (Fig. 5A–F), indicating that the exogenous IL-24 gene harbored in ZD55-IL-24 viral genome was most likely to play no role in B16-bearing immunocompetent mouse model. Similarly, our further data revealed that the anti-melanoma efficacy of ZD55-IL-24 had no significant enhancement compared with ZD55 and ZD55-EGFP (Supplementary Figs. 7A–C and 8A–E), further supporting the conclusion that the exogenous IL-24 gene played no role in B16-bearing immunocompetent mouse model.

### ZD55-IL-24 inhibits tumor growth in B16-bearing immunocompetent mouse model also through anti-angiogenesis pathway

**Fig. 7:**
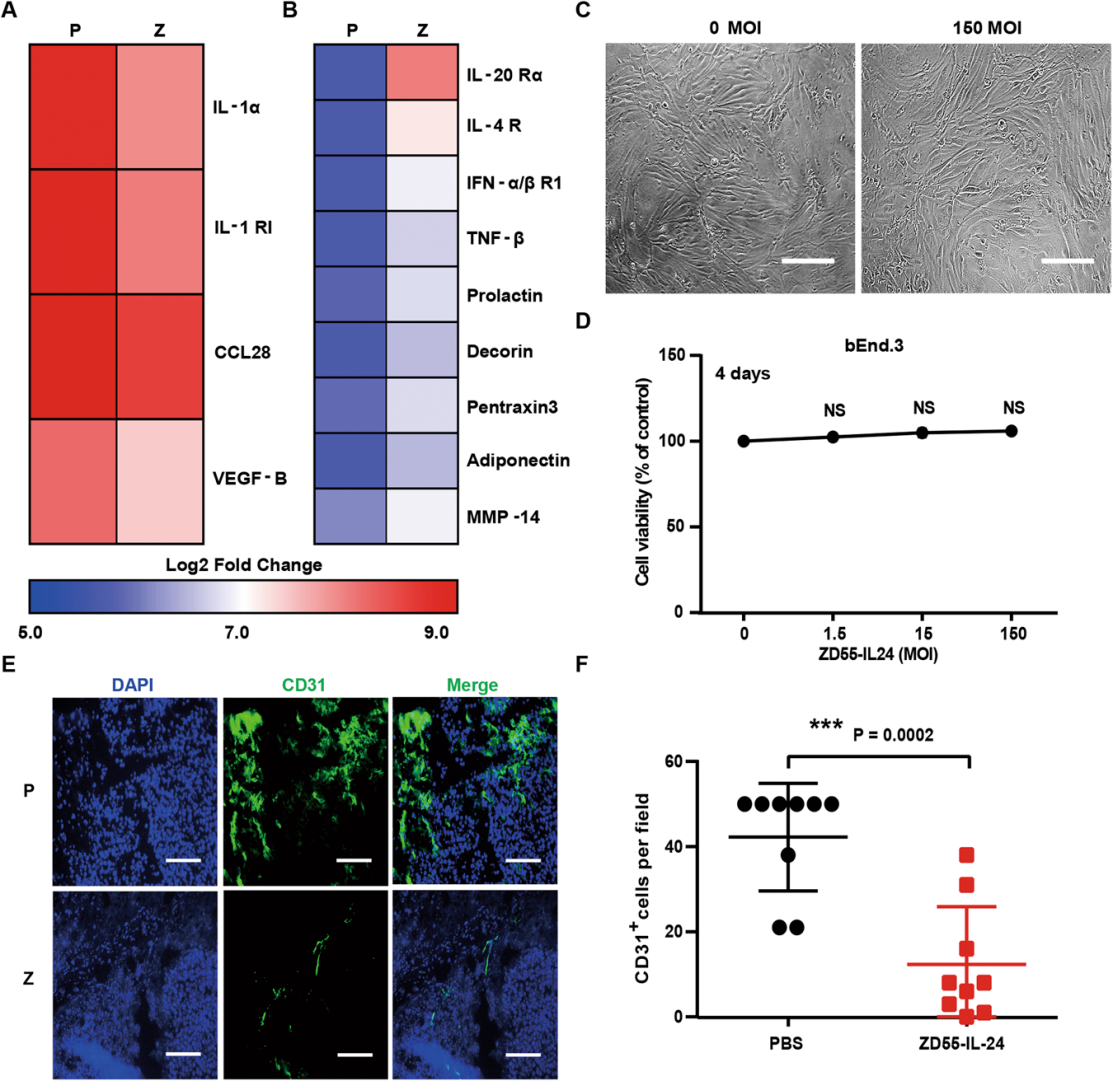
ZD55-IL-24 inhibits melanoma growth in B16-bearing immune-competent mouse model also through inhibiting angiogenesis. **A**, **B** Changes of the angiogenesis-related cytokines in tumors treated with ZD55-IL-24. **A** Heat-map of the down-regulated pro-angiogenic cytokines in Fig. 2C. **B** Heat-map of the up-regulated anti-angiogenic cytokines in Fig. 2D. **C**, **D** The anti-angiogenic effect of ZD55-IL-24 in vitro. **C** The murine vascular endothelial bEnd.3 cells were infected with ZD55-IL-24 at a series of MOI (PFU/cell) from 0 to 150, the appearance of cytopathic effect was monitored under microscope, and representative photographs were taken at day 4 post-infection **D** and cell viability was measured by CCK-8 assay. Results represent mean ± SEM of triplicate experiments and are expressed as a percentage of control cells. **E**, **F** The anti-angiogenic effect of ZD55-IL-24 in vivo. Tumors resected from B16-bearing C57BL/6 mice receiving PBS or ZD55-IL-24 treatment indicated in Fig. 1A were analyzed 2 days after the last injection by immunohistochemical staining. **E** Representative images of tumor sections immunostained for the endothelial marker CD31 (green) to label the blood vessels in tumors. **F** Quantification of the CD31^+^ cells in **E** (*n* = 9). Nuclei is counterstained with DAPI (blue). P PBS, Z ZD55-IL-24. Scale bars, 300 µm. Data are mean ± SD. Shown is one of three independent experiments.

Through analysis of the above down-regulated and up-regulated cytokines (Fig. 2A–D), we also noticed that 4 down-regulated cytokines had a function of promoting angiogenesis (Fig. 7A) and 9 up-regulated cytokines had a function of suppressing angiogenesis (Fig. 7B), hinting that ZD55-IL-24 might inhibit melanoma growth in B16-bearing immunocompetent mouse model also through inhibiting angiogenesis. To further determine whether the anti-angiogenesis pathway also played a role in B16-bearing immunocompetent mouse model, we next examined the direct killing effect of ZD55-IL-24 in the murine vascular endothelial cell line bEnd.3 in vitro. No significant cytotoxicity was observed in bEnd.3 cells (Fig. 7C, D), suggesting that ZD55-IL-24 could not inhibit angiogenesis in tumors through directly killing the vascular endothelial cells. We further assessed whether ZD55-IL-24 was able to inhibit angiogenesis in tumors using immunohistochemical analysis. Consistent with the cytokine antibody array results, the immunohistochemical results showed that the blood vessels in ZD55-IL-24-treated tumors were present at low density, whereas PBS-treated tumors produced more blood vessels (Fig. 7E, F), showing the anti-angiogenesis effects of ZD55-IL-24 in vivo. Therefore, these data showed that ZD55-IL-24 could also increase the levels of anti-angiogenic factors and decrease the levels of pro-angiogenic factors in tumors, which result in inhibition of angiogenesis, further contributing to the anti-melanoma efficacy of ZD55-IL-24 in B16-bearing immunocompetent mouse model.

### ZD55-IL-24 eradicates established melanoma in A375-bearing immunocompromised mouse model mainly through the direct pathway of inducing tumor-selective cytotoxicity, but not through the indirect pathway of inducing antitumor immunity and inhibiting angiogenesis

**Fig. 8:**
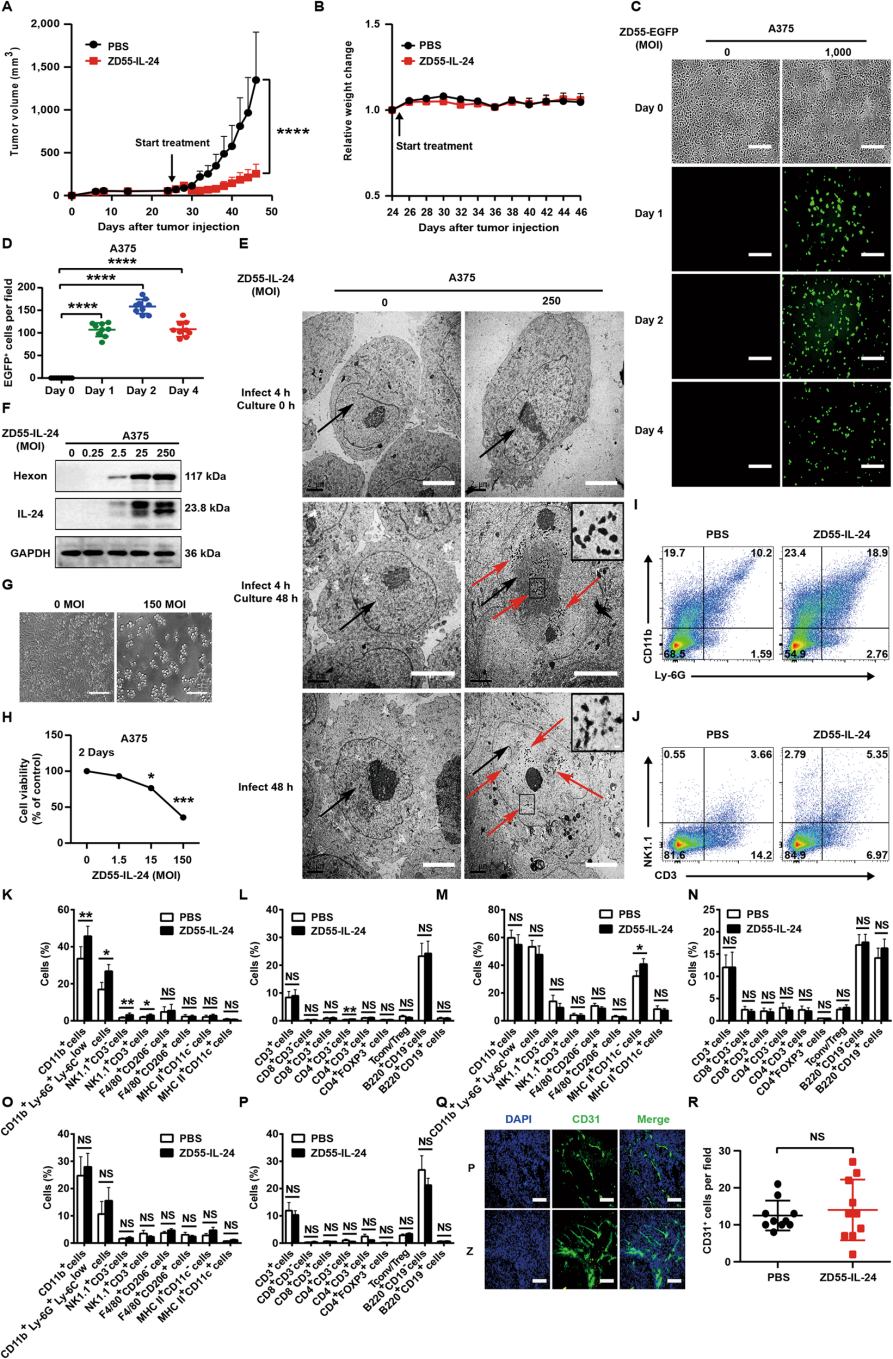
ZD55-IL-24 eradicates established melanoma in A375-bearing immunocompromised mouse model mainly through the classic direct killing pathway, but not through the antitumor immunity pathway and anti-angiogenesis pathway. **A**, **B** The antitumor efficacy of ZD55-IL-24 in A375-bearing immunocompromised mouse model. **A** Tumor growth curves over time for BALB/c nude mice inoculated with 2 × 10^6^ A375 cells s.c. in the right flank and treated with PBS or ZD55-IL-24 as indicated in Fig. 1A. **B** Body weight changes of the treated mice monitored during the therapy period. Data are presented as the mean ± SEM. *n* = 8 mice per group per experiment. **C**, **D** Fluorescence microscopic analysis of viral infection and exogenous gene expression in A375 cells. **C** The human melanoma A375 cells were infected with ZD55-EGFP at a MOI (PFU/cell) of 0 and 1000, and the viral infection and exogenous gene expression were monitored under the fluorescence microscope on Day 0, Day 1, Day 2, and Day 4 after infection. **D** quantification of the EGFP-positive A375 cells in **C** (*n* = 9). Error bars indicate mean ± SD. Shown is one of three independent experiments. Scale bars, 300 µm. **E** Representative transmission electron microscopy images of A375 cells treated with ZD55-IL-24 at a MOI (PFU/cell) of 0 and 250. Shown is one of three independent experiments. Nuclei and viral particles are indicated by the black and red arrow, respectively. Scale bar: 4 μm. Inset: high-power view, Scale bar: 100 nm. **F** Western blot analysis of viral infection and exogenous IL-24 expression in A375 cells infected with ZD55-IL-24 at a series of MOI (PFU/cell) as indicated. Shown is one of three independent experiments. **G**, **H** The cytotoxicity of ZD55-IL-24 in A375 cells in vitro. **G** The A375 cells at a density of 10^4^ cells/well cultured in 96-well plates were infected with ZD55-IL-24 at a series of MOI (PFU/cell) from 0 to 150, the appearance of cytopathic effect was monitored under microscope, and representative phase-contrast images were taken at 2 days later, and **H** cell viability was examined by CCK-8 assay. Scale bars, 300 µm. Results represent mean ± SEM of triplicate experiments and are expressed as a percentage of control cells. **I**–**P** Flow-cytometric analysis of immune cells infiltration in tumors, and recruitment and activation in spleens. Tumors and spleens resected from A375-bearing BALB/c nude mice receiving PBS or ZD55-IL-24 treatment indicated in Fig. 4A were analyzed by flow cytometry. **I** Shown are representative flow cytometry plots of tumor-infiltrating total myeloid cells and neutrophils in right tumors. **J** Representative flow cytometry plots of tumor-infiltrating NK cells and NKT cells in right tumors. **K** Percentages of innate immune cells in right tumors. **L** Percentages of adaptive immune cells in right tumors. **M** Percentages of innate immune cells in spleens. **N** Percentages of adaptive immune cells in spleens. **O** Percentages of innate immune cells in left tumors. **P** Percentages of adaptive immune cells in left tumors, mean ± SEM is shown. Data represent cumulative results from seven to eleven (**I**–**L**), eight to nine (**M**, **N**) or nine (**O**, **P**) independent experiments. **Q**, **R** The anti-angiogenic effect of ZD55-IL-24 in A375-bearing immunodeficient mouse model. Tumors resected from A375-bearing BALB/c nude mice receiving PBS or ZD55-IL-24 treatment indicated in Fig. 4A were analyzed by immunohistochemical staining. **Q** Representative images of tumor sections immunostained for the endothelial marker CD31 (green) to label the blood vessels in tumors. **R** Quantification of the CD31^+^ cells in **Q** (*n* = 10). Nuclei is counterstained with DAPI (blue). P PBS, Z ZD55-IL-24. Scale bars, 300 µm. Data are mean ± SD. Shown is one of two independent experiments.

Since nude mice lack a functional immune system due to a genetic mutation that caused a deteriorated or absent thymus, the antitumor efficacy of ZD55-IL-24 in immunocompromised mouse–human tumor xenograft models should rely mainly on direct killing pathway rather than antitumor immunity pathway. To further evaluate the contribution of direct killing pathway, we thus proceeded to investigate the anti-melanoma efficacy of ZD55-IL-24 in human A375 melanoma nude mouse model. Our data suggested that ZD55-IL-24 also induced robust tumor inhibition in A375-bearing immunocompromised mouse model (Fig. 8A, B), indicating that the classic direct killing pathway could also play an important role in tumor rejection. To determine whether the antitumor efficacy of ZD55-IL-24 in A375-bearing immunocompromised mouse model depended mainly on the direct killing pathway rather than antitumor immunity pathway, we first examined the viral infection and exogenous IL-24 expression in A375 cells in vitro. Our fluorescence microscopy, transmission electron microscopy, and Western blot results demonstrated the successful viral infection and production of exogenous IL-24 in A375 cells (Fig. 8C–F). Indeed, our CCK-8 results demonstrated that ZD55-IL-24 could directly kill A375 cells in vitro (Fig. 8G, H). These results suggested that ZD55-IL-24 was able to inhibit melanoma growth in A375-bearing nude mouse model via direct killing pathway.

As expected, our flow-cytometric results indicated that the antitumor immunity pathway played only a minor role in A375-bearing immunocompromised mouse model, although it could also induce innate antitumor immunity in local tumors (Fig. 8I–P). Consistent with the flow-cytometric results, our further results showed that ZD55-IL-24 delayed only the growth of local tumors rather than distant tumors (Supplementary Fig. 9A–F), confirming that ZD55-IL-24 merely induced local rather than systemic antitumor immunity in A375-bearing immunocompromised mouse model. Moreover, although it could inhibit melanoma growth in B16-bearing immunocompetent mouse model through anti-angiogenesis pathway, the anti-angiogenesis pathway had no contribution in A375-bearing immunocompromised mouse model (Fig. 8Q, R). Collectively, these data suggested that the observed therapeutic effect of ZD55-IL-24 in A375-bearing immunocompromised mouse model mainly depended on direct killing pathway, but depended little on the antitumor immunity pathway as well as anti-angiogenesis pathway.

## Discussion

Despite several reports have appeared in recent years showing the effects of oncolytic viruses on inhibiting the growth of tumors in immunocompetent mouse model and inducing the systemic antitumor immunity, the tumor cell lines used in these studies are the cell lines in which oncolytic viruses are able to successfully infect and directly lyse^14,22,23^. Thus, it is unable to understand the contribution of the direct killing pathway alone or the antitumor immunity pathway alone to the overall therapeutic efficacy of oncolytic viruses in these models. It is generally thought that the direct killing pathway plays a major role in tumor regression, while the antitumor immunity pathway plays only a minor role. Our current findings, however, indicate that the antitumor immunity pathway can also result in robust tumor growth inhibition (Fig. 1A–D) and even can result in small established tumor eradication (Supplementary Fig. 3A–E), suggesting that the antitumor immunity pathway can also play a very important role in cancer therapy. As best I can tell, this is the first report in which oncolytic virus has also been shown to be effective in an immunocompetent mouse model when the oncolytic virus is unable to successfully infect and directly lyse tumor cells. This finding provides us with an ideal tumor model for the investigation of antitumor immunity pathway induced by oncolytic viruses, because we have demonstrated that the direct killing pathway had no contribution in this model, and hence the interference of direct killing pathway is avoided.

Following cell death, the infected immunogenic tumor cells can release TAA, PAMPs, DAMPs, and cytokines, which lead to the generation of antitumor immunity^5,16,24^. Thus, it is thought that the successful viral infection and tumor cell death are central to induce antitumor immunity by oncolytic viruses^5,14–16,24^. However, our current results indicate that the antitumor immunity still can be induced even though ZD55-IL-24 cannot successfully infect and lyse tumor cells, suggesting that the antitumor immunity inducing process of ZD55-IL-24 is independent of successful viral infection and tumor cell death. ZD55-IL-24 utilizes a different mechanism to induce antitumor immunity in B16-bearing immunocompetent mouse model.

It is well-known that tumor cells tend to be self-origin, and are considered as “self” by original host immune system, hence it tend not to induce antitumor immunity in the body under normal circumstances^21,25,26^. ZD55-IL-24 as an oncolytic virus tend to be considered as “nonself” by original host immune system, hence it is very easy to establish the immune responses against ZD55-IL-24 in the body^27–29^. The immune system discriminate “self” from “nonself” based on subtle alterations in peptides displayed in association with MHC molecules at the cell surface^30,31^. Down-regulation of expression of MHC alleles, as well as tumor-specific antigens, is observed frequently during tumor progression, resulting in an impairment of tumor-specific immunity^32–34^. Remarkably, once internalized into the cytosol of tumor cells, the virions of ZD55-IL-24 are transported toward lysosomes where the viral particles are dismantled, and ultimately release the fragment of viral structural proteins and viral DNA^11^. The viral DNA then expressed the viral proteins such as E1A and replicated in tumor cells. The fragment of viral structural or expressed proteins and/or viral DNA will then be bound in the cytosol and displayed on the surface of infected tumor cells via MHC molecules, etc^20^. Our results, indeed, demonstrated that the surface MHC molecules (MHC I) and co-stimulatory molecules (CD80) of B16 cells were up-regulated by ZD55-IL-24 (Fig. 6G–L), which was also observed in oncolytic Newcastle disease virus^14^ and oncolytic reovirus^35^. The “non-self” viral antigen epitopes were indeed displayed on the surface of B16 cells (Fig. 6M). The B16 cells, therefore, are turned from the “self” state which is hard to be recognized by immune system into the “nonself” state which is easy to be recognized by immune system, enabling the immune system to recognize the tumor cells which are unrecognizable under normal circumstances. Together, our data show that ZD55-IL-24 can induce antitumor immunity in B16-bearing immunocompetent mouse model largely due to its ability to turn the tumor cells from the “self” state into the “nonself” state, but not due to its ability to lyse tumor cells and release TAAs, PAMPs, DAMPs as well as cytokines. Unfortunately, it is still unclear which component of ZD55-IL-24 is the “nonself” antigen epitopes and how they are presented on the surface of tumor cells in this study. Nonetheless, our data showed that the UV-inactivated ZD55-IL-24 that has the ability of viral attachment and entry, but has a damaged viral DNA, failed to induce antitumor immunity (Fig. 6A–E), indicating that the action of ZD55-IL-24 viral DNA (such as E1A expression or viral DNA replication) is likely to play a vital role in this process. Further investigation to this end is warranted.

In our studies, we find that ZD55-IL-24 induces almost identical tumor immune infiltration in both local and distant tumors (Fig. 4B–K, R–AA). However, the anti-melanoma efficacy of ZD55-IL-24 in local tumors is far higher than distant tumors in B16-bearing immunocompetent mouse model (Supplementary Fig. 2A–D). This phenomenon was also observed in oncolytic Newcastle disease virus^14^. However, it is unable to be well explained using the previous mechanism^5^, but can be well explained by our current mechanism. Although ZD55-IL-24 induces almost identical tumor immune infiltration in both local and distant tumors, the immune system is in fact unable to recognize the tumor cells in distant tumors for the lack of ZD55-IL-24 to turn the tumor cells from the “self” state into the “nonself” state.

Many studies have shown that the presence of tumor immune infiltration was a favorable prognostic indicator in a number of cancers^14,19,36^. Our data showed that ZD55-IL-24 can induce substantial immune cell infiltration in both local (Fig. 4B–K) and distant tumors (Fig. 4R–AA), as well as recruitment and activation of these cells in spleens (Fig. 4L–Q). NK cells and CD8^+^ T cells play a pivotal role in tumor regression^37,38^. NK cells play an early role, but the CD8^+^ T cells are required for long-term tumor control^14^. Indeed, depletion of NK1.1^+^ cells and CD8^+^ cells abrogates the therapeutic efficacy (Supplementary Fig. 5A–D), demonstrating ZD55-IL-24 can activate the tumor-infiltrating NK cells and CD8^+^ T cells, which then kill the tumor cells. In addition, important roles for other immune effector cells have also been described. Especially in the presence of tumor-opsonizing antibodies, NK cells, NKT cells, neutrophils, and macrophages can all contribute to direct antibody-dependent phagocytosis, reactive-oxygen-mediated cytotoxicity against tumors, and secretion of inflammatory cytokines and chemokines^19^. Depletion of neutrophils also abrogates the therapeutic efficacy, despite not statistically significant (Supplementary Fig. 5A–D), demonstrating ZD55-IL-24 can also activate the tumor-infiltrating neutrophils, which help tumor regression as well. However, macrophage depletion did not affect efficacy (Supplementary Fig. 5A–D). This result must be interpreted with caution because anti-CSF-1R depletion also results in concurrent depletion of M2 macrophages, which dampens antitumor immune responses^39^. Although there are no evidences to show that B cells and plasma cells contribute to cancer immunotherapy, they may help immune system rejecting tumors by producing tumor-opsonizing antibodies. During the priming of antitumor immunity, help signals are relayed from CD4^+^ T cells to CD8^+^ T cells by specific DCs to optimize the magnitude and quality of the CTL response^40^. Hence, CD4^+^ T cells help priming the antitumor immunity. Depletion of CD4^+^ cells indeed abrogates the therapeutic efficacy (Supplementary Fig. 5A–D), demonstrating ZD55-IL-24 can activate the tumor-infiltrating CD4^+^ T cells, which help tumor rejection. Although APCs, especially DCs, cannot produce direct tumor cell killing effects, these cells are central for the initiation of antigen-specific immunity^21^. Manipulation of APCs holds great potential for inducing efficient endogenous antitumor immunity^41^. ZD55-IL-24 can substantially increase the density of APCs in both local and distant tumors as well as spleens, indicating that ZD55-IL-24 can be used as an effective tool to manipulate APCs and initiate endogenous antitumor immunity for cancer immunotherapy. Notably, although there is a significant increase in the percentages of T_reg_ cells which suppressed tumor-specific immune responses, there is also a substantial increase in the percentages of T_conv_ cells, with marked enhancement of the CD4 effectors to T_reg_ ratios, which has been previously demonstrated to be a marker of a favorable immunological response^14,42^. Collectively, our data suggest that ZD55-IL-24 treatment can greatly promote tumor immune infiltration and recognition in B16-bearing immunocompetent mouse model with contributions to the induction of endogenous antitumor immunity.

ZD55-IL-24 treatment can greatly promote tumor immune infiltration in B16-bearing immunocompetent mouse model (Fig. 4A–K, R–AA). Therefore, the tumor-infiltrating immune cells are likely to produce large amounts of cytokines in tumors. Indeed, ZD55-IL-24 elicits substantial remodeling of the cytokine microenvironment in tumors, including the cytokines involved in antitumor immunity pathway (IL-2, IL-2 Rα, and B7-1, etc.), anti-angiogenesis pathway (L-20 Rα, TNF-β, IL-1α, etc.) and Wnt signaling pathway (Dkk-3, Kremen-2, Soggy-1, etc.) (Fig. 2A–D). Interleukins, which promote development and differentiation of leukocytes^43^, are one of the most remodeled cytokines involved in antitumor immunity pathway, such as IL-2, IL-5, IL-9, etc. Meanwhile, ZD55-IL-24 also up-regulates the expression of interleukin receptors (IL-2 Rα, IL-2 Rβ, IL-4 R, etc.) and costimulatory molecules (CD30, CD40, CD80, etc.), to further promote the antitumor immunity pathway. Chemokines, which direct the migration of leukocytes throughout the body^44^, are another large group of remodeled cytokines involved in antitumor immunity pathway, such as CCR6, CXCL16, CCL7, etc. Most importantly, ZD55-IL-24 therapy can result in substantial increase of effector molecules (TRAIL, TNF-β, Granzyme B, etc.), which directly induce the death of tumor cells in tumors, indicating the good prognosis of ZD55-IL-24 therapy. Notably, ZD55-IL-24 treatment leads to great increase of IL-9 up to 54 times. IL-9 is produced by a subset of activated CD4^+^ T cells and it induces the activation of epithelial cells, B cells, eosinophils, and mast cells^43^, suggesting the high IL-9 expression induced by ZD55-IL-24 is probably caused by the antiviral immune responses against ZD55-IL-24 because the target B cells mainly play role in antiviral immunity. We thus speculate that the highly produced IL-9 may contribute little to the therapeutic effect of ZD55-IL-24 in B16-bearing immunocompetent mouse model.

Interestingly, we notice that many down-regulated and up-regulated angiogenesis-related cytokines also function as immune-related cytokines (Fig. 7A, B), indicating that the anti-angiogenesis effect of ZD55-IL-24 in B16-bearing immunocompetent mouse model is most likely caused by the immune-related cytokines produced during the induction of antitumor immunity. This hypothesis is supported by the fact that the anti-angiogenesis effect of ZD55-IL-24 is only observed in B16-bearing immunocompetent mouse model (Fig. 7A–F) rather than A375-bearing immunodeficient mouse model (Fig. 8Q, R), because ZD55-IL-24 can induce robust systemic antitumor immunity and produce a large amount of cytokines only in B16-bearing immunocompetent mouse model rather than A375-bearing immunodeficient mouse model.

IL-24 is a unique member of the IL-10 gene family that displays direct antitumor activity through induction of autophagy and cancer-specific apoptosis, with no harmful effects toward normal cells or tissues^45^. It also induces indirect antitumor activity through stimulation of an antitumor immune response, inhibition of angiogenesis, and sensitization of cancer cells to radiation-induced, chemotherapy-induced, and antibody-induced killing^45^. Our previous data have demonstrated that the exogenous IL-24 gene harbored in the viral genome of ZD55-IL-24 was able to be highly expressed in many kinds of human tumor cells and played an important role in immunocompromised mouse–human tumor xenograft models^7,8^. However, our current results indicate that ZD55-IL-24 cannot successfully infect and express exogenous IL-24 gene in B16 cells (Fig. 5A–F), and the transgene IL-24 has no significant contribution to the antitumor efficacy of ZD55-IL-24 in B16-bearing immunocompetent mouse model (Supplementary Fig. 8A–E), suggesting the transgene IL-24 in fact plays no role in B16-bearing immunocompetent mouse model. Indeed, it is well known that almost all murine cells and tissues are not supportive of human adenovirus replication and gene expression. Our data are in line with the published reports. ZD55-IL-24 can infect, directly lyse, and express a large amount of exogenous IL-24 in human cancer cells within cancer patient’s tumors. Thus, ZD55-IL-24 can produce a large amount of exogenous IL-24 in patients and then the produced IL-24 can induce autophagy and cancer-specific apoptosis in patients. In addition, the immunostimulatory effect of the transgene IL-24 can also be observed in patients since patient’s immune system is intact. We, therefore, believe that the transgene IL-24 is likely to play an important role in patients, despite it plays no role in B16-bearing immunocompetent mouse model.

According to our study, we now know that the overall anti-melanoma efficacy of ZD55-IL-24 in patients should be contributed by the direct killing pathway, antitumor immunity pathway, IL-24-mediated other antitumor pathway, and anti-angiogenesis pathway (Supplementary Fig. 10). The direct killing pathway plays a decisive role in A375-bearing immunodeficient mouse model, while playing no role in B16-bearing immunocompetent mouse model for the inability of successful viral infection and exogenous IL-24 expression in B16 cells. Conversely, the antitumor immunity pathway plays a decisive role in B16-bearing immunocompetent mouse model, while playing a minor role in A375-bearing immunodeficient mouse model for the lack of a functional immune system in immunodeficient mice. Indeed, although ZD55-IL-24 can also induce innate antitumor immunity in local tumors (Fig. 8I–P), the antitumor immunity pathway in fact contributes little to the overall anti-melanoma efficacy of ZD55-IL-24 in A375-bearing immunodeficient mouse model (Figs. 1A–D, 3K–M). IL-24 works only in A375-bearing immunodeficient mouse model, but not in B16-bearing immunocompetent mouse model, because it is highly expressed in A375 cells but not in B16 cells. Moreover, IL-24 is still unable to exert its maximal efficacy in A375-bearing immunodeficient mouse model, albeit with high expression, because its immunostimulatory effect cannot work. Hence, the contributions of exogenous IL-24 gene harbored in ZD55-IL-24 viral genome have yet to be fully understood at this time. The anti-angiogenesis pathway plays a minor role in B16-bearing immunocompetent mouse model (Figs. 7A–F, 3K–M, 1A–D), while playing no role in A375-bearing immunodeficient mouse model (Fig. 8Q, R). Since cancer patient’s immune system is intact, and ZD55-IL-24 can infect, directly lyse and express exogenous IL-24 in human cancer cells within patient’s tumors, we thus speculate that all the antitumor pathways are probably able to act synergistically in patients. The findings of this study can help us to understand the antitumor mechanism of ZD55-IL-24 and other oncolytic adenovirus in patients.

## Supplementary information

Supplementary figure legends

Supplementary Figure 1

Supplementary Figure 2

Supplementary Figure 3

Supplementary Figure 4

Supplementary Figure 5

Supplementary Figure 6

Supplementary Figure 7

Supplementary Figure 8

Supplementary Figure 9

Supplementary Figure 10

Supplementary Table 1
