# Supplementary material for: The armed oncolytic adenovirus ZD55-IL-24 eradicates melanoma by turning the tumor cells from the self-state into the nonself-state besides direct killing
Source: Cell Death Dis. 2020 Nov 30;11(11):1022. doi: 10.1038/s41419-020-03223-0 (PMC7705698; doi:10.1038/s41419-020-03223-0)
Supplement: Supplementary file 1 — Supplementary figure legends [file 41419_2020_3223_MOESM1_ESM.docx]

**Supplementary figure legends**

**Supplementary Figure 1** Assessment the direct killing effect of ZD55-IL-24 in various murine and their corresponding human tumor cell lines *in vitro*. **(A)** The murine lung cancer LLC1 cells and **(C)** the human lung cancer A549 cells at a density of 10^4^ cells/well cultured in 96-well plates were infected with ZD55-IL-24 in a wide range of MOI (PFU/cell), the appearance of cytopathic effect was monitored under microscope, and representative photographs were taken at 2 days later, and **(B, D)** cell viability was examined by CCK-8 assay. **(E)** The murine colon carcinoma CT26.WT cells and **(G)** the human colon carcinoma SW620 cells at a density of 10^4^ cells/well cultured in 96-well plates were infected with ZD55-IL-24 in a wide range of MOI (PFU/cell), the appearance of cytopathic effect was monitored under microscope, and representative photographs were taken at 3 days later, and **(F, H)** cell viability was examined by CCK-8 assay. Scale bars, 300 µm. Results represent Mean ± SEM of triplicate experiments and are expressed as a percentage of control cells.

**Supplementary Figure 2** ZD55-IL-24 has antitumor efficacy not only on local tumors but also on distant tumors in B16-bearing immunocompetent mouse model. B16-bearing C57BL/6 mice were treated with PBS or ZD55-IL-24 as indicated in **Figure 4A**, starting when the average right tumor volume was about 80 mm^3^. **(A)** Growth of local and **(B)** distant tumors. **(C)** Weight of local and **(D)** distant tumors resected from the sacrificed mice at the end of the experiment. **(E)** Overall survival. **(F)** Body weight changes of mice monitored during the therapy period. Data represent results from one of two independent experiments with n = 10 per group. Mean ± SEM is shown.

**Supplementary Figure 3** ZD55-IL-24 therapy cures small established tumors and establishes protective memory in B16-bearing immunocompetent mouse model. C57BL/6 mice were inoculated with B16 tumors and treated with PBS or ZD55-IL-24 as indicated, and the surviving animals were injected with 2 × 10^5^ B16 cells in the right flank on day 90 and followed for tumor re-challenge rejection. **(A)** Treatment scheme. **(B)** Tumor growth curves. **(C)** Overall survival. **(D)** Body weight changes of the treated mice monitored during the therapy period. **(E)** The fraction of long-term survivors that rejected a re-challenge with 2 × 10^5^ B16 cells on day 110. n = 3 C57BL/6 mice per group for PBS, and n = 8 for ZD55-IL-24. s.c., subcutaneous injection. i.t., intratumoral injection. Data are presented as Mean ± SEM.

**Supplementary Figure 4** Gate strategy of Flow cytometric analysis. **(A)** Shown is representative gating for immune cells immunostained with surface marker. **(B)** Shown is representative gating for T_conv_ and T_reg_.

**Supplementary Figure 5** Antitumor activity of ZD55-IL-24 in B16-bearing immunocompetent mouse model depends on CD8^+^ T cells, CD4^+^ T cells, NK cells, and neutrophils. Groups of C57BL/6 mice were inoculated with 10^6^ B16 cells s.c. in the right flank**.** Cellular subsets were depleted by administering 400 µg of depleting antibody i.p. twice weekly beginning one day prior to initiation of ZD55-IL-24 therapy following the timeline of **Figure 4A**. Depletions included CD8^+^ T cells with anti-CD8α ; CD4^+^ T cells with anti-CD4; NK cells with anti-NK1.1; neutrophils with anti-Ly-6G; and macrophages via blockade of CSF-1R, which was depleted using 300 µg of anti-CSF-1R every other day beginning one day prior to ZD55-IL-24 treatment. (**A)** Confirmation of depletions for CD8^+^ T cells, CD4^+^ T cells, NK cells, neutrophils, and macrophages from PBMCs on day 15. Shown is one representative of three independent experiments. **(B)** Tumor growth curves. **(C)** Overall survival. **(D)** Body weight changes of the treated mice monitored during the therapy period. Data represent results from one of two independent experiments with n = 5 per group. Mean ± SEM is shown.

**Supplementary Figure 6** Cytotoxicity identification of UV or HTHP inactive ZD55-IL-24 *in vitro* and *in vivo*. **(A, B)** Cytotoxicity identification of UV or HTHP inactive ZD55-IL-24 *in vitro*. **(A)** The human melanoma A375 cells at a density of 10^4^ cells/well cultured in 96-well plates were infected with ZD55-IL-24, UV inactive ZD55-IL-24, or HTHP inactive ZD55-IL-24 at a MOI (PFU/cell) of 0, 1,000 and 10,000, the appearance of cytopathic effect was monitored under microscope, and representative photographs were taken at 4 days later, and **(B)** cell viability was examined by CCK-8 assay. Mean ± SD is shown. Results are expressed as a percentage of control cells. Scale bars, 300 µm. **(C-F)** Cytotoxicity identification of UV or HTHP inactive ZD55-IL-24 *in vivo.* BALB/c nude mice were inoculated with A375 tumors and treated with PBS, ZD55-IL-24, UV inactive ZD55-IL-24, or inactive ZD55-IL-24 as indicated in **Figure 4A** (n = 10 mice per group). **(C)** *In vivo* tumor growth curves. **(D)** Weight of tumors resected from the sacrificed mice at the end of the experiment. **(E)** Photograph of tumors resected from the sacrificed mice at the end of the experiment. **(F)** Body weight changes of mice monitored during the therapy period. UV, ultraviolet. HTHP, high temperature and high pressure. E, Eradication. Mean ± SEM is shown.

**Supplementary Figure 7** Identification of various adenoviruses. **(A)** Identification of ZD55-EGFP using Fluorescence microscopy. **(B)** Identification of Ad5.WT and ZD55 using PCR analysis. **(C)** Identification of ZD55-IL-24 using Western blot analysis.

**Supplementary Figure 8** The exogenous IL-24 gene harbored in ZD55-IL-24 viral genome has no significant contribution to the antitumor efficacy of ZD55-IL-24 in B16-bearing immunocompetent mouse model. C57BL/6 mice were inoculated with B16 tumors and treated with PBS or various adenoviruses as indicated in **Figure 4A**. **(A)** In vivo tumor growth curves. **(B)** Photographs of tumors resected from the sacrificed mice at the end of the experiment. **(C)** Weight of tumors resected from the sacrificed mice at the end of the experiment. **(D)** Overall survival. **(E)** Body weight changes of mice monitored during the therapy period. E, Eradication. D, Death. Data represent results from one of two independent experiments with n = 10 per group. Mean ± SEM is shown.

**Supplementary Figure 9** ZD55-IL-24 has remarkable anti-melanoma efficacy only on local tumors, but not on distant tumors in A375-bearing immunocompromised mouse model. A375-bearing BALB/c nude mice were treated with PBS or ZD55-IL-24 as indicated in **Figure 4A**, starting when the average right tumor volume was about 80 mm^3^. **(A)** Growth of local and **(B)** distant tumors. **(C)** Weight of local and **(D)** distant tumors resected from the sacrificed mice at the end of the experiment. **(E)** Overall survival. **(F)** Body weight changes of mice monitored during the therapy period. Data represent results from one of two independent experiments with n = 10 per group. Mean ± SEM is shown.

**Supplementary Figure 10** Mechanisms of ZD55-IL-24-mediated anti-melanoma effect. ZD55-IL-24 utilizes several mechanisms to kill melanoma, including the direct killing pathway, antitumor immunity pathway, IL-24-mediated other antitumor pathway and anti-angiogenesis pathway. **(A)** Mechanism of ZD55-IL-24 viral vector-mediated tumor-selective cytotoxicity. Once inside the tumor cells, ZD55-IL-24 expresses several gene products that target cellular proteins and modulate various cellular processes, such as preventing apoptosis or inducing cell-cycle entry. These promote viral replication and production of viral proteins, eventually leading to tumor cell lysis and viral progeny release. The viral progeny then spread throughout a tumor, infect and lyse surrounding cancer cells, further producing more viral progeny and improving the anti-melanoma efficacy of ZD55-IL-24. **(B)** Mechanism of ZD55-IL-24 viral vector-induced systemic antitumor immunity. Once attaching to and entering tumor cells, ZD55-IL-24 puts a “nonself” label into tumor cells which are recognized as “self” by the original host immune system, and then the tumor cells are turned from the state of “self” into the state of “nonself”, enabling them easy to be recognized by the immune system. Subsequently, a systemic non-specific and specific antitumor immunity is spontaneously induced by ZD55-IL-24, ultimately leading to the inhibition of both local and distant tumors. **(C)** Mechanisms of the exogenous IL-24-mediated anti-melanoma effect. The IL-24 gene harbored in the viral genome of ZD55-IL-24 can replicate as the viral genome replication and express a large amount of IL-24 in tumor cells. The produced IL-24 can then be released from the infected tumor cells to the vicinity of uninfected tumor cells and transported to the whole body through blood and lymphatic circulation, exerting its anti-melanoma effects in the whole body through inducing cancer-selective cytotoxicity, stimulating a systemic antitumor immune response, inhibiting angiogenesis and mediating other anti-melanoma effects. **(D)** Mechanism of ZD55-IL-24 viral vector-mediated anti-angiogenesis effect. ZD55-IL-24 can also stimulate the body to produce various anti-angiogenic cytokines and inhibit the production of various pro-angiogenic cytokines in tumors, resulting in the inhibition of angiogenesis in tumors and ultimately inhibiting the growth of melanoma as well. The direct killing pathway, which includes the ZD55-IL-24 viral vector-mediated tumor-selective cytotoxicity and the IL-24-mediated tumor-selective cytotoxicity, plays a decisive role in A375-bearing immunodeficient mouse model, while playing no role in B16-bearing immune-competent mouse model. On the contrary, the antitumor immunity pathway, which includes the ZD55-IL-24 viral vector-induced systemic antitumor immunity and the IL-24-induced systemic antitumor immunity, plays a decisive role in B16-bearing immune-competent mouse model, while playing a minor role in A375-bearing immunodeficient mouse model. The IL-24-mediated other antitumor pathway, which includes sensitization of cancer cells to radiation-, chemotherapy- and antibody-induced killing, plays a minor role in A375-bearing immunodeficient mouse model, while playing no role in B16-bearing immune-competent mouse model. The anti-angiogenesis pathway, which includes the ZD55-IL-24 viral vector-mediated anti-angiogenesis effect and the IL-24-mediated anti-angiogenesis effect, plays a minor role in B16-bearing immunocompetent mouse model, while playing no role in A375-bearing immunodeficient mouse model. It is unable to observe the anti-melanoma efficacy of antitumor immunity pathway in A375-bearing immunocompromised mouse model for the lack of a functional immune system; Meanwhile, it is unable to observe the anti-melanoma efficacy of direct killing pathway and the IL-24-induced systemic antitumor immunity in B16-bearing immunocompetent mouse model, because ZD55-IL-24 can’t successfully infect, directly kill, and express exogenous IL-24 in B16 cells. For these reasons, it is currently unable to observe the synergistic effect of all the antitumor pathways utilized by ZD55-IL-24 in pre-clinical study. However, the immune systems of cancer patients are intact, and ZD55-IL-24 can infect and kill cancer cells directly in cancer patients, and it also can express exogenous IL-24 in patient’s tumors, we therefore speculate that all the antitumor pathways utilized by ZD55-IL-24 are probably able to act synergistically in patients.
