# Supplementary figures and images for: The armed oncolytic adenovirus ZD55-IL-24 eradicates melanoma by turning the tumor cells from the self-state into the nonself-state besides direct killing
Source: Cell Death Dis. 2020 Nov 30;11(11):1022. doi: 10.1038/s41419-020-03223-0 (PMC7705698; doi:10.1038/s41419-020-03223-0)

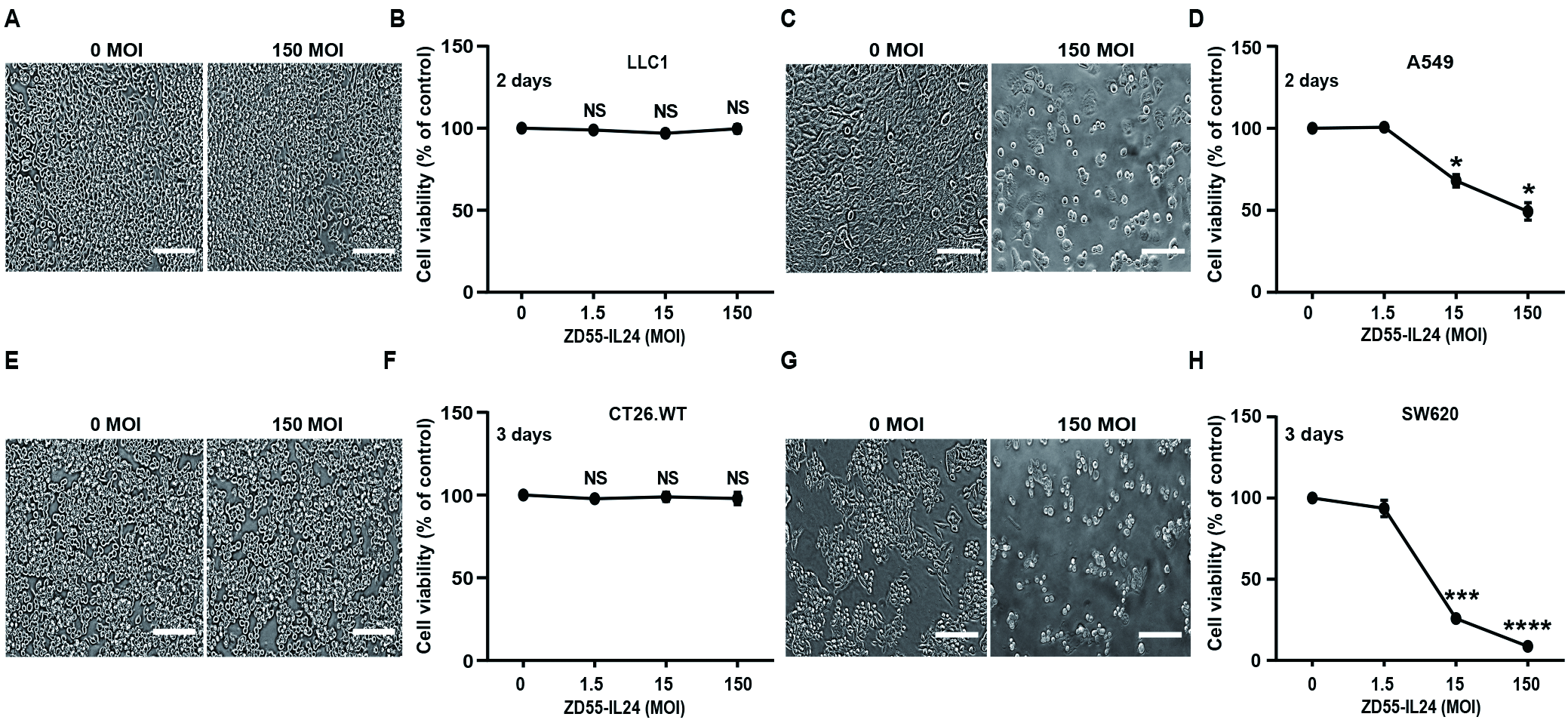

Supplement: Supplementary file 2 — Supplementary Figure 1 [file 41419_2020_3223_MOESM2_ESM.tif]

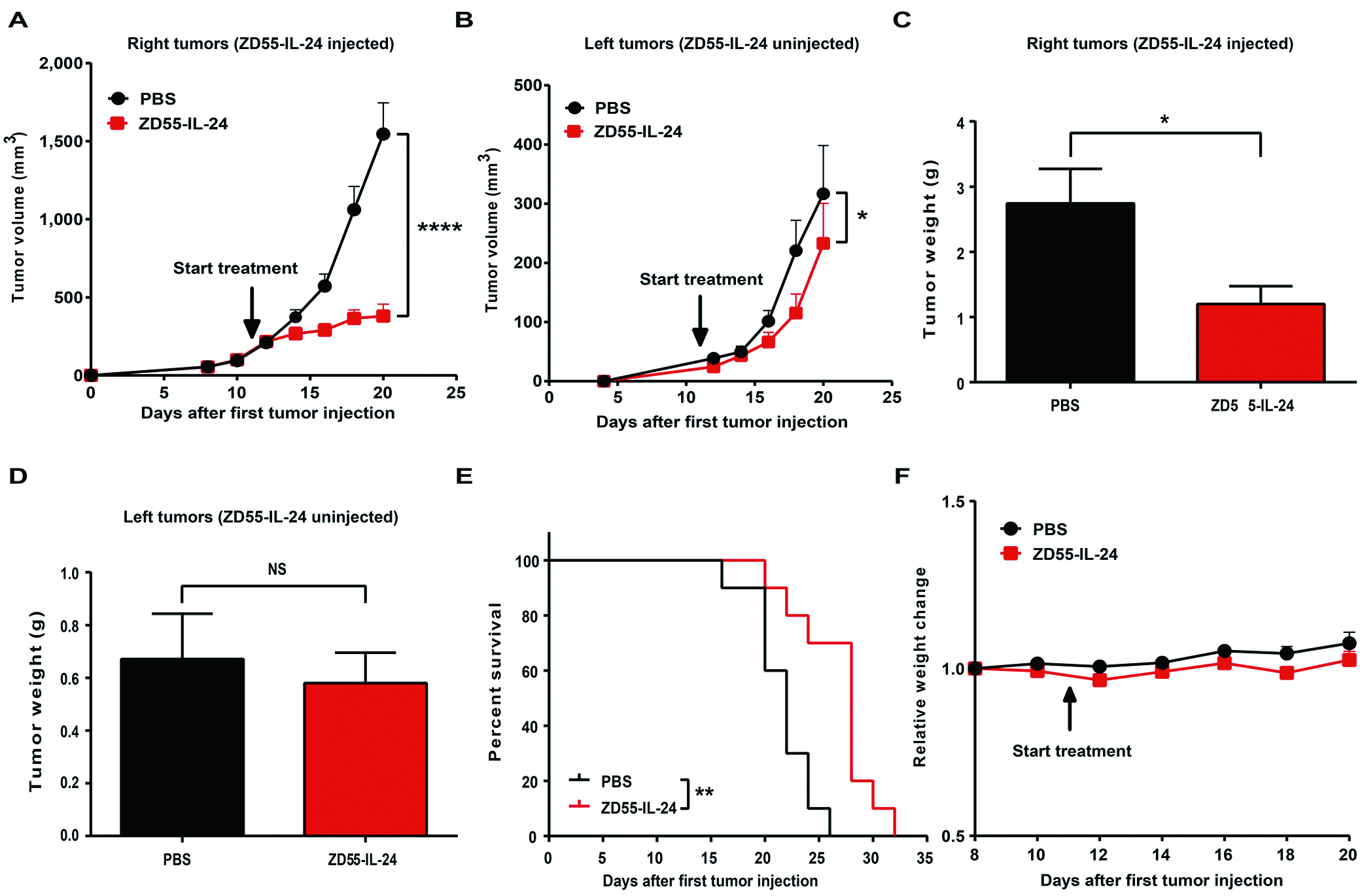

Supplement: Supplementary file 3 — Supplementary Figure 2 [file 41419_2020_3223_MOESM3_ESM.tif]

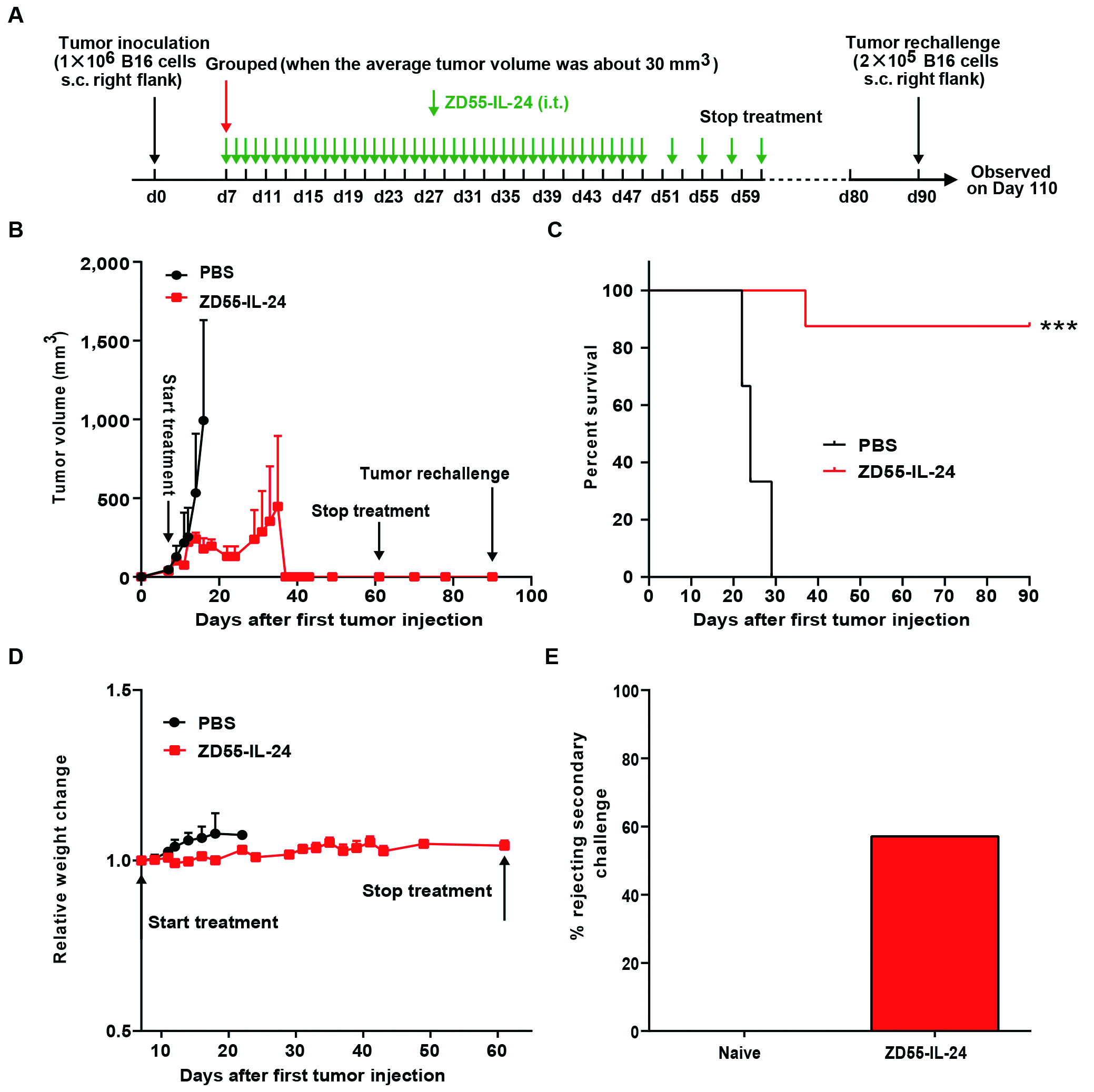

Supplement: Supplementary file 4 — Supplementary Figure 3 [file 41419_2020_3223_MOESM4_ESM.tif]

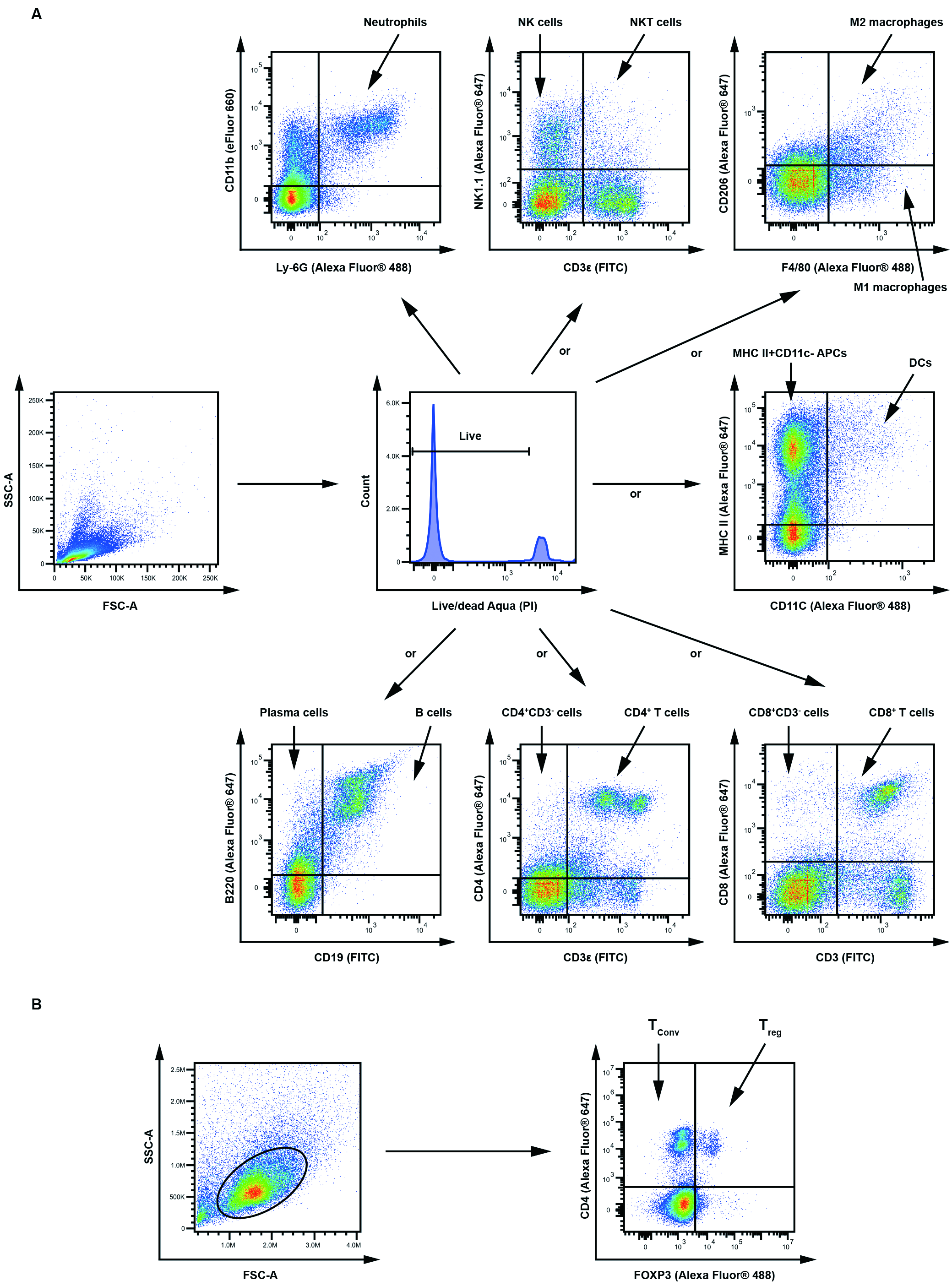

Supplement: Supplementary file 5 — Supplementary Figure 4 [file 41419_2020_3223_MOESM5_ESM.tif]

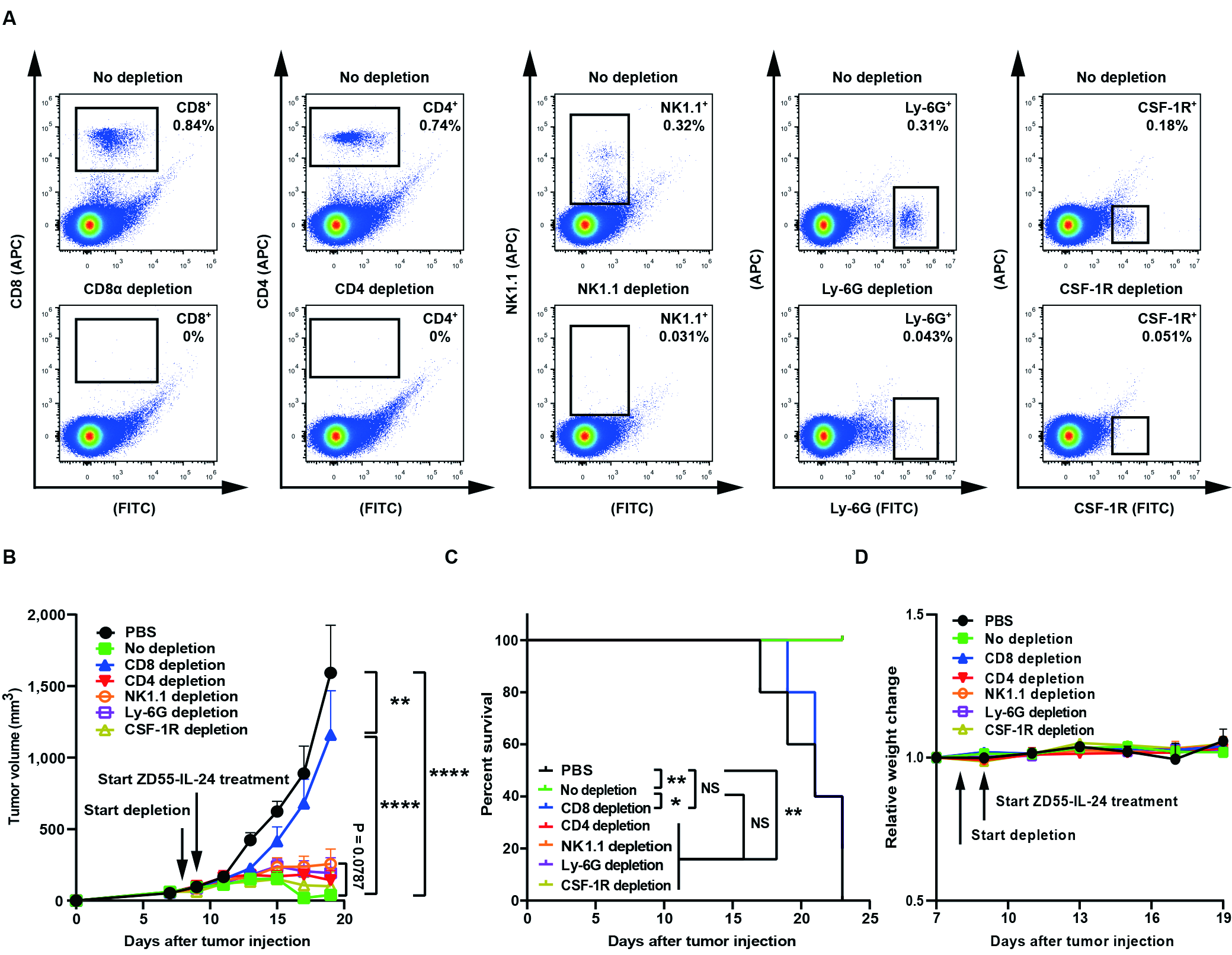

Supplement: Supplementary file 6 — Supplementary Figure 5 [file 41419_2020_3223_MOESM6_ESM.tif]

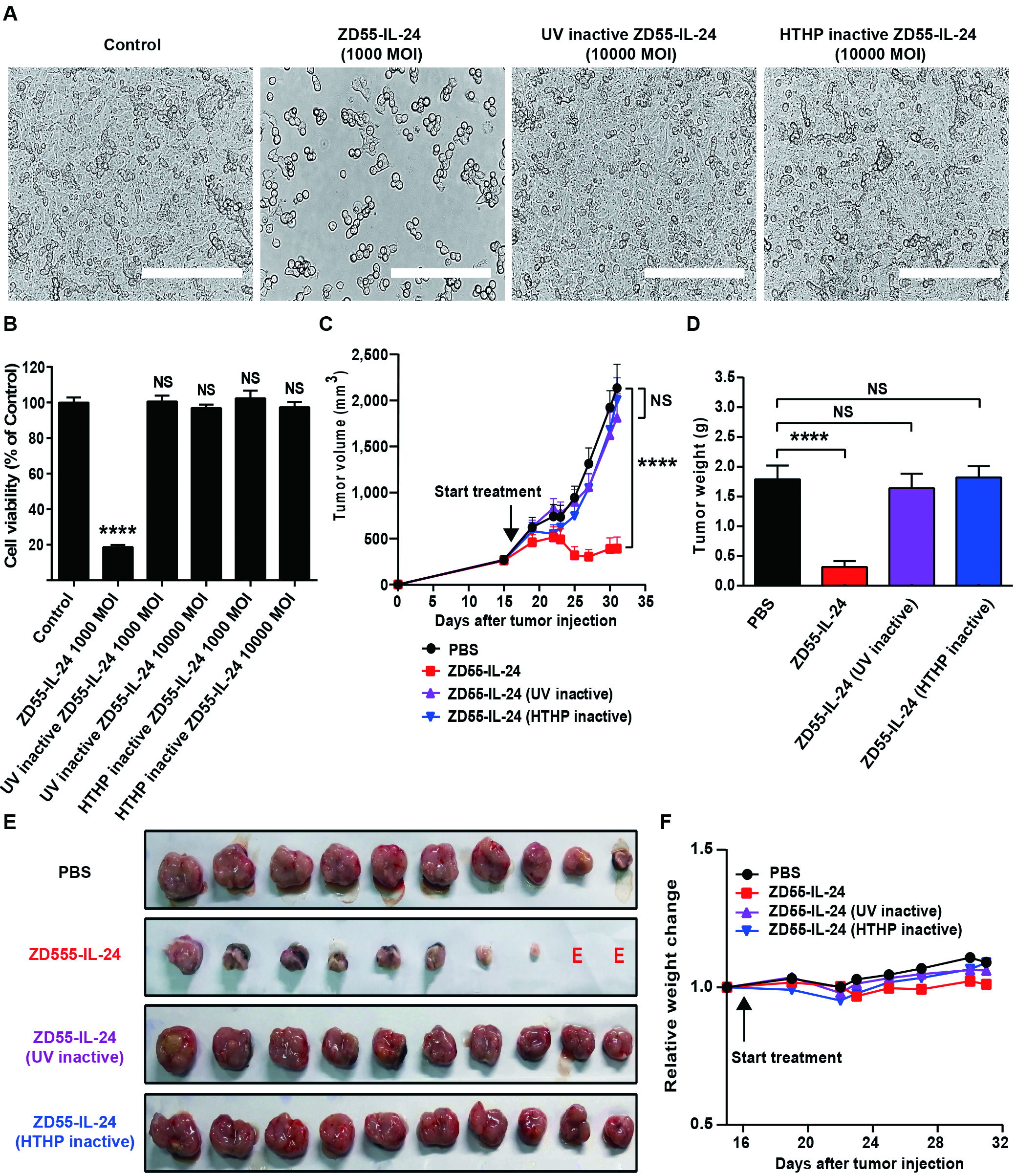

Supplement: Supplementary file 7 — Supplementary Figure 6 [file 41419_2020_3223_MOESM7_ESM.tif]

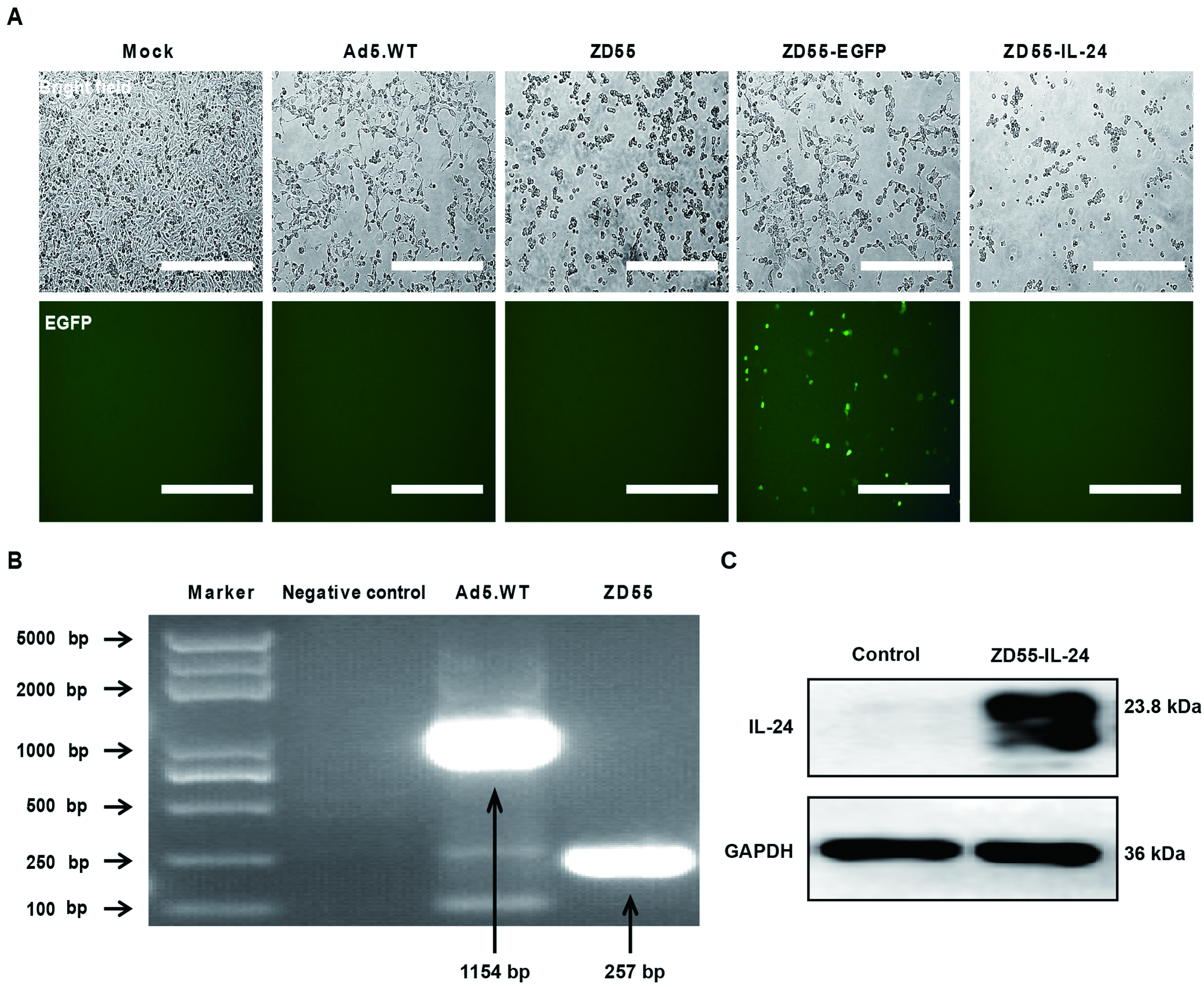

Supplement: Supplementary file 8 — Supplementary Figure 7 [file 41419_2020_3223_MOESM8_ESM.tif]

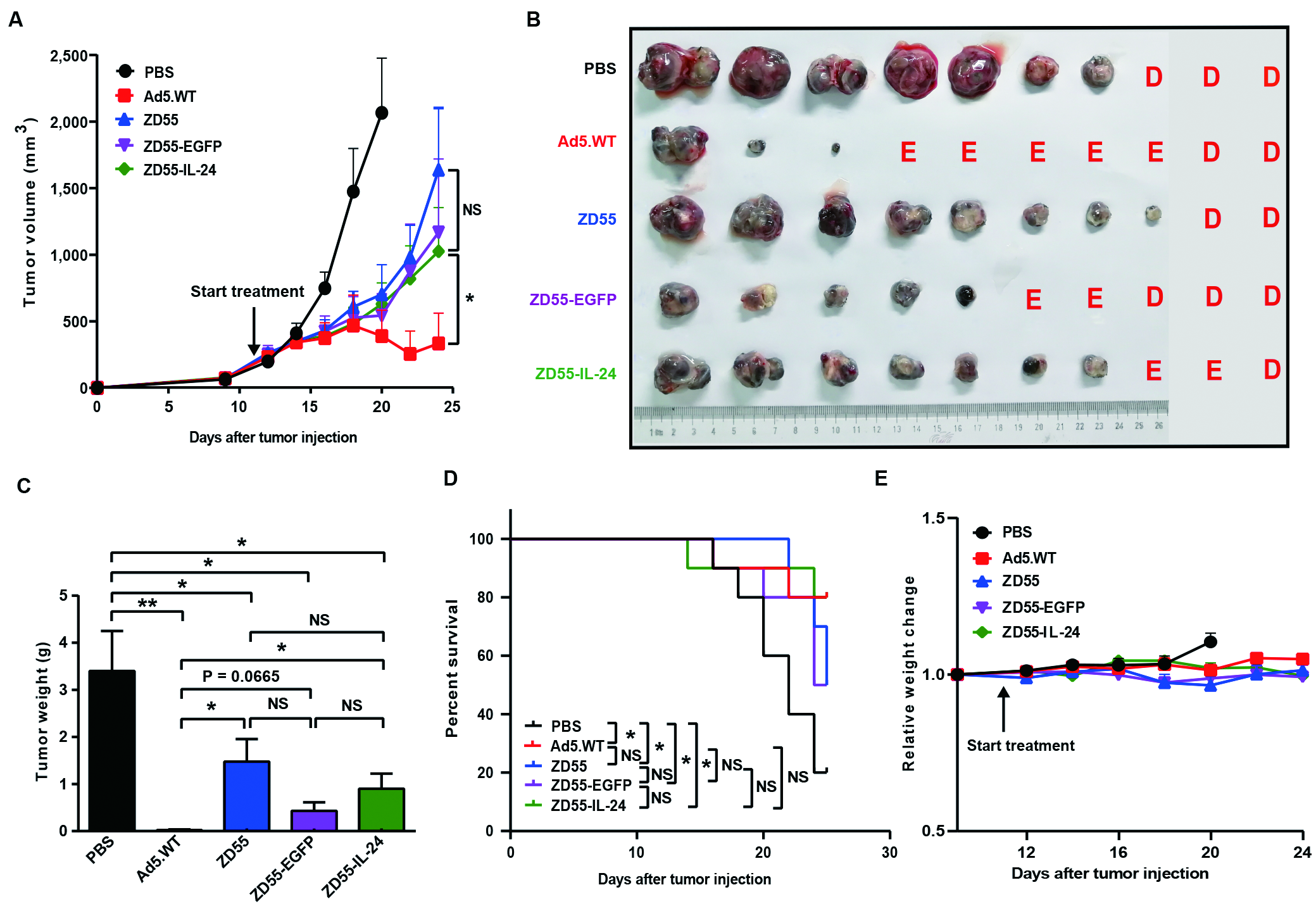

Supplement: Supplementary file 9 — Supplementary Figure 8 [file 41419_2020_3223_MOESM9_ESM.tif]

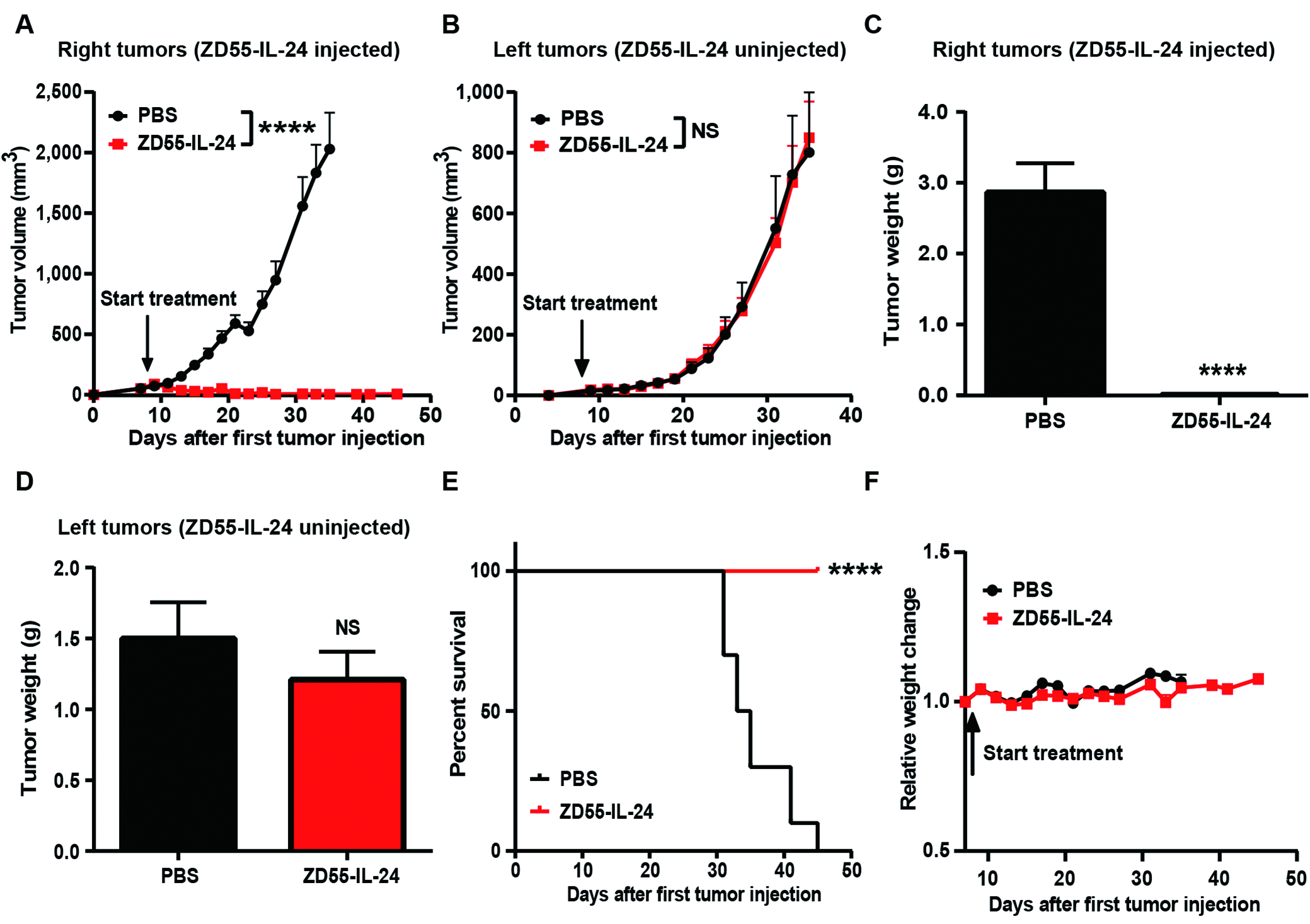

Supplement: Supplementary file 10 — Supplementary Figure 9 [file 41419_2020_3223_MOESM10_ESM.tif]

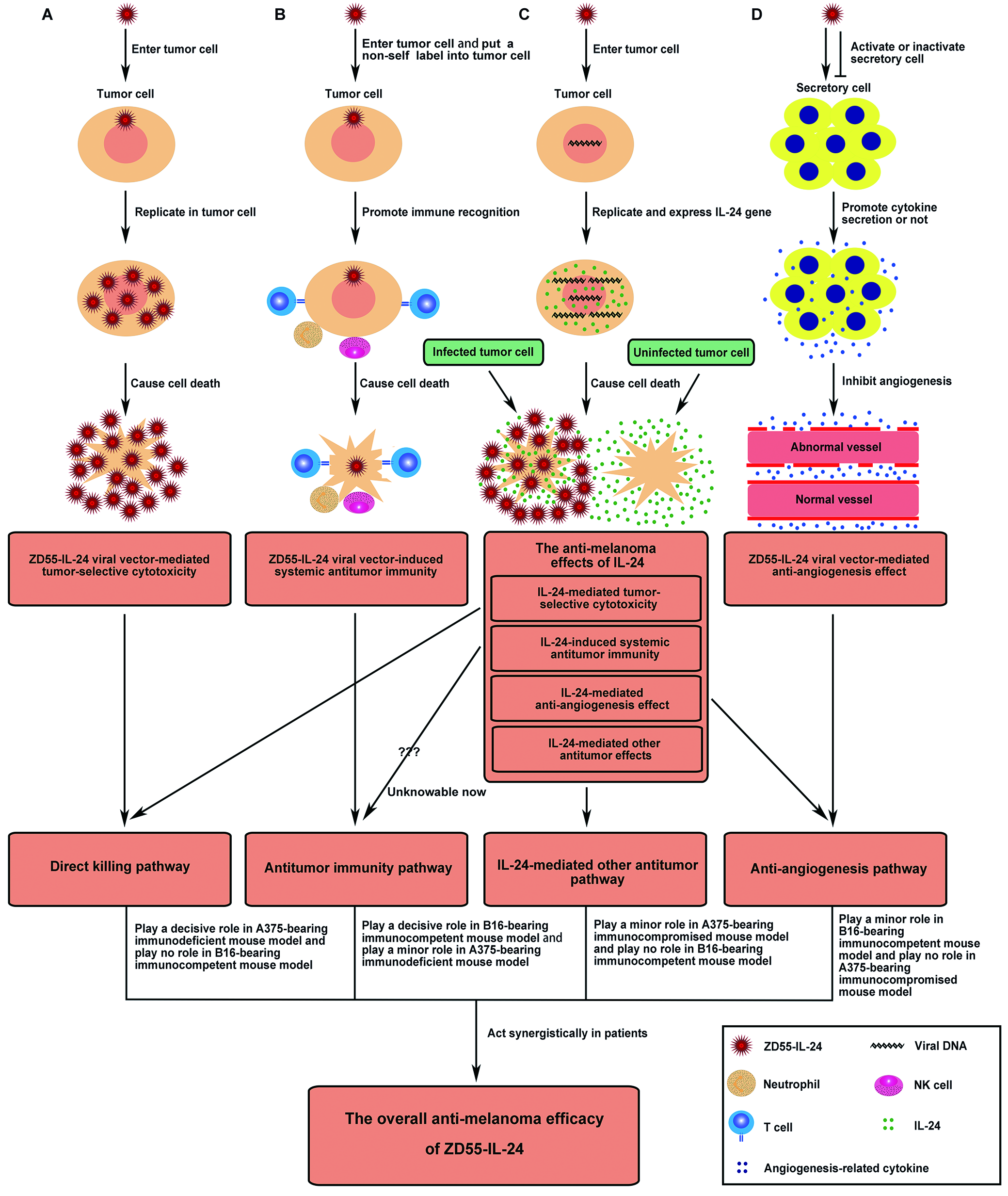

Supplement: Supplementary file 11 — Supplementary Figure 10 [file 41419_2020_3223_MOESM11_ESM.tif]
